# Occupational Contact Dermatitis in the Post-COVID Era: From Barrier Dysfunction and Microbiome Dysbiosis to Prevention and Precision Management

**DOI:** 10.3390/jcm15166353

**Published:** 2026-08-17

**Authors:** Laura Maghiar, Andrada Iftode, Teodor-Andrei Maghiar, Raul Chioibas, Titus Grecu, Carmen Neamțu, Sandor Mircea Ioan, Cristina-Adriana Dehelean, Cristina Dumitrescu, Andreea-Adriana Neamțu

**Affiliations:** 1 Department of Psycho-Neurosciences and Rehabilitation, Faculty of Medicine and Pharmacy, University of Oradea, Universității Str., No. 1, 410087 Oradea, Romania; laura.maghiar@uoradea.ro; 2 Department of Dermatovenerology, Clinical County Emergency Hospital Bihor, Gheorghe Doja Str., No. 65, 410169 Oradea, Romania; 3 Department of Toxicology, Faculty of Pharmacy, “Victor Babeș” University of Medicine and Pharmacy, Eftimie Murgu Square, No. 2, 300041 Timișoara, Romania; andradaiftode@umft.ro (A.I.); cadehelean@umft.ro (C.-A.D.); cristina.grosu@umft.ro (C.D.); andreea.neamtu@umft.ro (A.-A.N.); 4 Research Centre for Pharmaco-Toxicological Evaluation, Faculty of Pharmacy, “Victor Babeș” University of Medicine and Pharmacy, Eftimie Murgu Square, No. 2, 300041 Timișoara, Romania; 5 Department of Surgical Disciplines, Faculty of Medicine and Pharmacy, University of Oradea, Universității Str., No. 1, 410087 Oradea, Romania; msandor@uoradea.ro; 6 Department of Surgery, Pelican Hospital, Corneliu Coposu Str., No. 2, 410450 Oradea, Romania; 7 Department of Surgery I, Faculty of Medicine, “Victor Babeș” University of Medicine and Pharmacy, Eftimie Murgu Square, No. 2, 300041 Timișoara, Romania; 8 CBS Medcom Hospital, Popa Șapcă Str., No. 12, 300047 Timișoara, Romania; 9 Department of Plastic Surgery, The Christie NHS Foundation Trust, 550 Wilmslow Road, Manchester M20 4BX, UK; titus.grecu@nhs.net; 10 Faculty of Dentistry, “Vasile Goldiș” Western University of Arad, Liviu Rebreanu Str., No. 86, 310045 Arad, Romania; neamtu.carmen@uvvg.ro; 11 Department of Surgery I, Clinical County Emergency Hospital of Arad, Andrenyi Karoly Str., No. 2–4, 310037 Arad, Romania; 12 CF Oradea Clinical Hospital, Republicii Str., No. 56, 410159 Oradea, Romania; 13 Department of Pathology, Clinical County Emergency Hospital of Arad, Andrenyi Karoly Str., No. 2–4, 310037 Arad, Romania; 14 Department of Pathology, “Pius Brînzeu” Clinical County Emergency Hospital, Liviu Rebreanu Boulevard, No. 156, 300723 Timișoara, Romania

**Keywords:** occupational contact dermatitis, hand eczema, skin barrier, skin microbiome, antimicrobial peptides, emollients, healthcare workers, prevention, delgocitinib

## Abstract

**Background/Objectives:** Occupational contact dermatitis (OCD) is the most common work-related skin disease, accounting for roughly 90–95% of occupational dermatoses and falling predominantly on the hands. It is rarely dangerous yet imposes a substantial burden through impaired quality of life, lost productivity, and premature exit from affected trades. The COVID-19 pandemic intensified this burden among healthcare workers, in whom the pooled one-year prevalence of self-reported hand eczema reaches around 27%; meta-analytic data link the increased risk principally to frequent handwashing and wet work rather than to alcohol-based hand rub. **Methods:** This narrative review, which follows a non-systematic, thematically organised search strategy rather than PRISMA methodology, integrates current evidence on the epidemiology, pathophysiology, diagnosis, prevention, and management of OCD, with particular emphasis on the self-reinforcing cycle linking skin barrier disruption, microbiome dysbiosis, and antimicrobial-peptide dysregulation to inflammation. **Results:** We critically appraise the prevention evidence, foregrounding the low certainty of the existing trial base and the tension between the randomised trials of primary and secondary prevention, which have been null, and the encouraging but uncontrolled results of structured tertiary-prevention programmes. We summarise recent therapeutic advances, including topical delgocitinib, and situate the field within the World Health Organisation’s 2025 recognition of skin diseases as a global public health priority. Established evidence and hypotheses are kept separate throughout: we additionally advance, explicitly as a conjecture rather than as a demonstrated mechanism, a conceptual trans-kingdom dialogue model in which protease-generated LL-37 fragments may modulate staphylococcal quorum sensing, and each step of that model is labelled according to whether the supporting evidence is direct, extrapolated, or as yet untested. **Conclusions:** We argue that the prevention failure is less one of biology than of trial design and measurement, and outline the research needed to close the gap.

## 1. Introduction

Occupational contact dermatitis (OCD) is the most common work-related skin disease, accounting, in Western countries, for roughly 90–95% of all occupational skin disease and falling, in the great majority of cases, on the hands [1]. About four in five cases are irritant in nature and the remainder allergic, although the two forms overlap clinically and frequently coexist on the same pair of hands, so that patch testing serves to identify or exclude a relevant contact allergy rather than to resolve the two into mutually exclusive categories [2]. The condition is rarely life-threatening, yet its impact is anything but trivial: chronic hand eczema impairs quality of life, disrupts work, and drives substantial costs, with mean total annual societal costs per patient ranging from €1813 to €7738, up to 57% of patients taking sick leave and up to 25% reporting job loss or a change in job [3]. The scale of the underlying burden has now been recognised at the highest level of global health governance. In May 2025, the Seventy-eighth World Health Assembly adopted resolution WHA78.15, formally designating skin diseases as a global public health priority and calling for the development of a global action plan on skin diseases [4]. Occupational dermatoses, as the most frequent work-related skin conditions, sit squarely within that mandate.

Quantifying the problem at the population level reinforces the point. A recent analysis of the Global Burden of Disease 2021 data estimated 241 million prevalent cases of dermatitis worldwide in 2021, a 38.8% rise since 1990, alongside 405 million incident cases in that year [5]. These figures refer to dermatitis as an aggregated category and are not disaggregated by subtype, so they bound rather than quantify the contact dermatitis burden specifically. Behind these figures lies a recurring pattern of underreporting: registry-based incidence consistently understates the true occupational burden by more than two orders of magnitude at the extremes when registry figures are set against cohort data, as set out in Section 3 [6]. Certain occupations carry a disproportionate share of risk—healthcare workers, hairdressers, metalworkers, food handlers, cleaners, and construction workers chief among them—and for many of these, hand eczema is a leading cause of job change or premature exit from the trade [1].

The COVID-19 pandemic sharpened this picture considerably, particularly for healthcare workers. Intensified hand hygiene, markedly increased handwashing frequency, and prolonged glove occlusion combined to place the skin of the hands under sustained stress. A 2024 systematic review and meta-analysis reported a pooled one-year prevalence of self-reported hand eczema of 27.4% (95% CI 19.3–36.5) among healthcare workers [7], against a pooled one-year prevalence of 9.1% (95% CI 8.4–9.8) in the general population [8]; the wide confidence interval around the healthcare-worker estimate reflects considerable heterogeneity between studies. Importantly, meta-analytic data associate the increased risk principally with repeated washing and wet work rather than with alcohol-based hand rub; this distinction carries direct implications for how prevention is framed [9]. The pandemic thus served as a large, involuntary natural experiment, exposing both the vulnerability of the skin barrier to occupational stressors and the limits of current preventive practice.

Two clarifications are needed before proceeding. First, we use the term post-COVID era throughout in a descriptive rather than a biological sense, to denote the period from 2022 onwards in which the acute phase of the pandemic had passed but its legacy persisted in three specific respects: durably altered hand-hygiene and glove-use behaviour among healthcare and other workers; revised institutional infection-control policies that outlasted the emergency itself; and the large body of epidemiological data generated during and immediately after the acute phase, on which much of the recent literature rests. The term carries no implication of a direct or persistent pathogenic effect of SARS-CoV-2 on the skin of the hands.

Second, the occupational picture developed here sits within a broader pandemic-era dermatological signal that is worth acknowledging even though it lies outside the review’s main focus. SARS-CoV-2 infection itself was associated with a spectrum of cutaneous manifestations, including maculopapular exanthems that could cluster within affected families [10] and, in Omicron-variant infection, wheals, erythema and papular or papulovesicular eruptions in which pruritus was the dominant symptom [11]; emergency-department data spanning the pre-pandemic and pandemic periods documented a concurrent rise in inflammatory dermatoses, with urticaria, atopic dermatitis and contact dermatitis prominent among them [12]. Occupational facial dermatoses attributable to personal protective equipment ran in parallel with the hand disease that concerns us here: prolonged mask wear was associated with acne, rosacea flares and perioral dermatitis in addition to pressure-related injury [13], and a systematic review of reactions to facial protective equipment in healthcare workers documented irritant, occlusive and allergic mechanisms operating at these sites [14]. The mechanisms are recognisably the same as those described below for the hands, namely occlusion, raised local pH, friction and altered surface ecology, and this parallelism is one reason for treating the pandemic as an occupational-dermatology natural experiment rather than a purely respiratory event.

A note on terminology is also necessary, because the relevant terms are not synonyms and have at times been used interchangeably in this literature. In this review, occupational contact dermatitis (OCD) denotes contact dermatitis at any body site for which occupational exposure is a substantial causal contributor; it subsumes irritant contact dermatitis (ICD) and allergic contact dermatitis (ACD) as mechanistic categories. Hand eczema is a morphological and topographical designation covering any eczematous dermatitis of the hands irrespective of cause. Occupational hand eczema is the subset of hand eczema to which work exposure contributes, and chronic hand eczema (CHE) is the subset persisting for more than three months or recurring twice or more within twelve months, the definition on which the therapeutic trials cited in Section 7 are based. Atopic hand eczema denotes hand involvement in a constitutionally atopic individual and is an endogenous rather than an occupational category, although it frequently coexists with occupational exposure and is a major susceptibility factor for it. Where evidence obtained in one of these populations is applied to another, we indicate this explicitly in the text.

That second point deserves emphasis, because it motivates much of what follows. Despite decades of clinical attention, the evidence underpinning prevention of occupational irritant hand dermatitis remains of low certainty. The 2018 Cochrane review concluded that barrier creams and moisturisers may help, but found no randomised trials of protective gloves and judged the effect of skin-protection education uncertain [15]. The two largest subsequent trials reported essentially null results on their primary outcomes, deepening rather than resolving the question of what actually works [16,17]. Meanwhile, advances in barrier biology, the skin microbiome, and innate immunity have begun to reshape our mechanistic understanding of how occupational dermatitis develops, and a new generation of targeted therapies has entered the clinic [18]. This review brings these threads together. We summarise the epidemiology and high-risk occupations; trace the mechanistic cascade linking wet work, irritants, and occlusion to barrier disruption, dysbiosis, and dysregulated innate immunity; outline current diagnosis; and critically appraise the evidence for prevention and management—with the aim of reconciling a rapidly advancing science with a prevention base that has, so far, lagged behind.

## 2. Materials and Methods

This article is a narrative review and does not follow a formal systematic-review protocol or PRISMA reporting; its aim was to integrate mechanistic, clinical, and preventive evidence on occupational contact dermatitis into a coherent synthesis rather than to answer a single narrowly framed question. Relevant literature was identified through searches of PubMed/MEDLINE, the Cochrane Library, Scopus, and Web of Science, supplemented by hand-searching of the reference lists of key articles and current clinical guidelines.

Search terms combined controlled vocabulary with free-text keywords—among them “occupational contact dermatitis,” “hand eczema,” “irritant” and “allergic contact dermatitis,” “skin barrier,” “filaggrin,” “skin microbiome,” “antimicrobial peptides,” “patch testing,” “prevention,” “emollients,” “gloves,” “healthcare workers,” and “delgocitinib”—used alone and in combination with Boolean operators. The search covered the period from 2000 to 2026 and was restricted to articles published in English.

**Figure 1:**
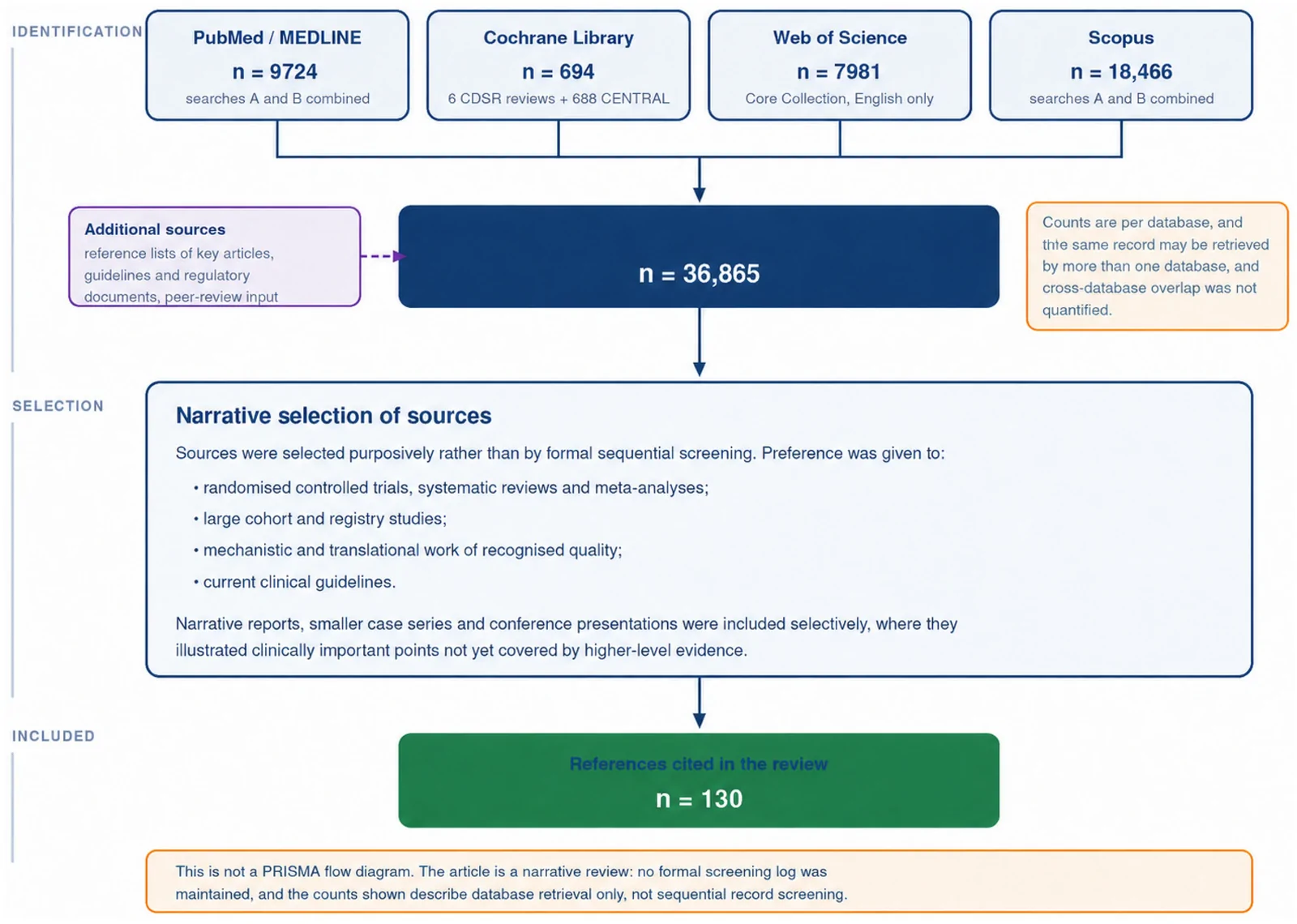
Flow of literature identification and selection. The upper row gives the number of records retrieved from each database using the strategies reported in Supplementary Materials S1; the total is the sum of the per-database combined counts and includes records retrieved by more than one database, cross-database overlap not having been quantified. Because this article is a narrative review, the retrieved records were not screened sequentially against predefined eligibility criteria, so the diagram records the passage from database retrieval to the final cited set and is not a PRISMA flow diagram. References identified by hand-searching the reference lists of key articles, together with clinical guidelines, regulatory documents and references suggested during peer review, entered the review outside the database searches and are not included in the totals shown.

To make the search reproducible, its parameters are reported here in full. The literature search was originally completed in June 2026 and was updated during revision in August 2026; the complete database-specific strategies, including the exact Boolean strings together with the field tags, subject headings and filters applied in each database, are provided as Supplementary Materials S1. The updated search retrieved 36,865 records in total: 9724 from PubMed/MEDLINE, 7981 from the Web of Science Core Collection, 18,466 from Scopus, and 694 from the Cochrane Library (6 Cochrane Reviews and 688 records in CENTRAL). Because this is a narrative rather than a systematic review, these records were not screened sequentially against predefined eligibility criteria, and no dual-independent screening, formal deduplication cascade or risk-of-bias assessment was undertaken. The search output was instead used to identify the candidate literature from which sources were selected purposively, according to the prioritisation criteria set out below and supplemented by hand-searching of the reference lists of key articles and current guidelines; selection was carried out by the first author and the resulting set was reviewed and agreed by the co-authors. The publications cited in this review therefore represent a deliberate, criterion-guided selection rather than the endpoint of a systematic screening process, and we state this explicitly so that the basis of the synthesis is transparent. The flow from database retrieval to the final reference set is summarised in Figure 1, which is offered as a descriptive account of identification and selection rather than as a PRISMA flow diagram, for the reasons just given. The distance between the number of records retrieved and the number ultimately cited follows from how the blocks were combined rather than from unusual selectivity: a deliberately broad condition block was crossed with an equally broad mechanism, diagnosis and intervention block, so that a substantial part of the yield concerned contact dermatitis outside the occupational setting, or the mechanistic terms as they arise in other inflammatory skin diseases.

Priority was given to randomised controlled trials, systematic reviews and meta-analyses, large cohort and registry studies, mechanistic and translational work of recognised quality, and current clinical guidelines. Narrative reports, smaller case series, and conference presentations were included selectively where they illustrated clinically important points—for example, protein contact dermatitis or the most recent therapeutic developments—not yet covered by higher-level evidence. Where the strength of the underlying evidence is limited, as is notably the case for the prevention literature, this is stated explicitly in the text rather than smoothed over.

Records identified were screened by title and abstract, and potentially relevant full-text articles were assessed against the prioritisation criteria above; the reference lists of included articles and guidelines were examined for additional sources. In keeping with the narrative scope, neither a formal risk-of-bias assessment nor quantitative pooling was undertaken; instead, the evidence was synthesised thematically around the central framework of a self-perpetuating barrier–microbiome–antimicrobial-peptide–inflammation loop, integrating mechanistic, clinical, and preventive findings. To make the underlying evidence base explicit, the design and principal findings of the key studies informing each thematic domain are summarised in Table 1.

## 3. Epidemiology and High-Risk Occupations

Among occupational diseases of the skin, contact dermatitis stands alone in frequency. It represents roughly 90–95% of all occupational dermatoses in Western countries and falls predominantly on the hands, the body site most directly and repeatedly exposed to the irritants, allergens, and wet work of the workplace [1]. The split between the two forms is consistent across case series: irritant contact dermatitis accounts for the large majority of cases, commonly cited at around 80%, with allergic contact dermatitis making up most of the remainder [2]. This distinction matters clinically, yet it is one that the bedside cannot reliably make. Irritant and allergic disease frequently coexist on the same pair of hands, and patch testing identifies or excludes a relevant contact allergy rather than assigning a case definitively to one category or the other. A negative patch test does not exclude a coexisting irritant mechanism, and a positive reaction still requires assessment of clinical and occupational relevance before it can be held responsible for the disease. Transcriptomic work supports this overlap directly: in strong and extreme allergic patch-test reactions in non-atopic patients, 26 of the 132 nickel-specific differentially expressed genes had been reported in earlier studies as irritant-related, leading the authors to conclude that contact allergy may be accompanied by some degree of irritancy and that the two processes can co-exist within a single reaction [28]. This is a point we return to in the section on diagnosis.

Putting firm numbers to incidence is harder than it should be. Registered occupational contact dermatitis occurs at roughly 0.5–1.9 cases per 1000 full-time workers per year and makes up as much as 30% of all notified occupational diseases, but these figures rest on notification systems that are known to capture only a fraction of true cases [2]. The gap becomes stark when registry data are placed beside cohort studies. In healthcare workers, for instance, a systematic review found registry-based incidence of 0.6–6.7 cases per 10,000 person-years against cohort-based incidence of 15.9–780.0 per 10,000 person-years—a difference in more than two orders of magnitude at the extremes, driven largely by apprentice nurses and dental personnel [6]. Underreporting, in other words, is not a minor caveat but a structural feature of the epidemiological record, and any reading of official statistics has to allow for it.

At the population level, the Global Burden of Disease 2021 study provides the broadest available frame. It estimated 241 million prevalent cases of dermatitis worldwide in 2021, a rise of 38.8% since 1990, alongside roughly 405 million incident cases and 8.18 million disability-adjusted life years [5]. The age-standardised prevalence rate edged down slightly over the same period, which suggests that the absolute increase is driven more by population growth and ageing than by rising underlying risk. Contact dermatitis is the most common subtype within this total, with approximately 253 million new cases in a single year [5]. Atopic dermatitis, while a distinct entity, is relevant here too: it carries the highest disability burden among skin diseases and represents a key endogenous risk factor for hand eczema, predisposing the barrier to breakdown under occupational stress [5].

The economic weight of the condition follows from its chronicity. A systematic review of chronic hand eczema costs reported mean total annual societal costs per patient ranging from roughly €1813 to €7738 in Europe, with the highest estimates concentrated in occupation-related disease [3]. Most of that cost is indirect: in European studies, lost productivity accounted for between half and seven-tenths of the total, reflecting absenteeism and reduced capacity at work rather than treatment expense alone [3]. More than one in five patients took sick leave, with mean reported absence ranging from about 7 to 35 days per year [3]. National data tell the same story from a different angle—in Germany, work-related hand eczema accounts for around 90% of all occupational-disease claims under the relevant statutory category [3].

Risk is not distributed evenly across the workforce. A handful of occupations carry a disproportionate share, and the pattern is remarkably stable across countries and decades. Hairdressers are among the hardest hit: in a Danish register-based study of more than five thousand graduates, 44.3% had left the trade after a mean of roughly eight years, and chronic hand eczema was substantially more common in those who had left than in those still working [19]. Metalworkers exposed to cutting fluids are a second classic high-risk group, in whom metal sensitisation compounds the irritant load: a meta-analysis of 29 studies and 5691 metalworkers found pooled contact allergy prevalences of 8.1% to cobalt, 8.5% to chromium and 13.5% to nickel [29]. Among cleaners, prevalence estimates span a wide range, from about 2% to 30%, reflecting heterogeneous exposures to wet work, bleach, and ammonia [30]. Construction and cement workers illustrate the allergic end of the spectrum: in a fifteen-year patch-test series, cement-induced chromate allergy presented at a younger median age than other chromate sensitivity, 32 against 42 years, and hand eczema was the clinical presentation in 88.9% of cases; the proportion of chromate-sensitive patients with clinically relevant cement exposure rose over the study period, from 7.7% in 2002–2004 to 28.7% in 2011–2013 [31]; that series comes from a national setting outside the scope of the European chromium(VI) directive considered in Section 6, which is the likely explanation for the contrast with the European trend. Tellingly, even low-irritant occupations carry measurable risk. In a meta-analysis of incidence by occupation, pooled rates per 100 person-years were 21.4 in hairdressers, 16.9 in nurses and 12.4 in metalworkers, against 4.9 in office workers; the office figure emerged only after three years in the occupation, there being no significant excess at one year, a reminder that low-level cumulative exposure is not the same as no exposure [32]. Table 2 summarises the principal irritant and allergen exposures across the highest-risk occupations.

One geographical gap is worth naming directly, because it bears on the contribution this review and its authors can make. Romania-specific prevalence data for occupational skin disease are, for practical purposes, absent from the peer-reviewed literature; the principal national publication is a questionnaire-development paper that itself notes the prevalence in Romania is unknown [33]. The reason is structural rather than accidental: a survey of key occupational health and safety informants across 22 Eastern European countries, conducted within the COST StanDerm action and including Romania, found that although national legislation is now largely harmonised with Western provisions, a sustained resistance to notifying occupational skin disease persists, and it identified the clinical, organisational and educational barriers responsible. Its authors make the point that concerns us here: where few cases are notified, there is no political impetus for change [34]. The nearest Eastern European datapoint comes from Poland, and it is a modest one: an online questionnaire study of 142 women, which found hand eczema to be more common in younger adults and identified disinfectant use during the pandemic as a key factor in exacerbating hand lesions [20]. The one-year prevalence figures of 11.5% in women and 6.7% in men that are sometimes quoted alongside that study are general-population meta-analytic estimates rather than Polish data [8], which underlines rather than relieves the regional shortfall. The scarcity of regional data is not merely an academic inconvenience. It means that prevention policy across much of Central and Eastern Europe is being shaped without a clear picture of local burden—a gap that future surveillance, including work originating from Romanian centres, is well placed to fill.

### Glove-Related Dermatitis After COVID-19

Few exposures illustrate the post-COVID shift in occupational skin disease as clearly as the glove. The pandemic produced an unprecedented and sustained rise in glove use among healthcare workers and, for a time, the general public, layered on top of intensified hand hygiene; the predictable consequence was a wave of hand dermatitis, and the elevated one-year prevalence among healthcare workers noted above reflects that combined burden [6,7]. Meta-analytic data attribute the increased risk principally to frequent handwashing and wet work rather than to alcohol-based hand rub [9]; the independent contribution of glove occlusion has not been quantified in those analyses, although the mechanism is well characterised. The dominant mechanism is irritant rather than allergic: prolonged occlusion traps sweat, raises the surface pH, macerates the stratum corneum, and impairs barrier recovery, so that the glove intended to protect the skin becomes, with enough hours of wear, a cause of disease in its own right.

A minority of cases are genuinely allergic. Contact allergy to rubber accelerators is the usual mechanism. A meta-analysis of 106 studies and 826,543 patch-tested patients found pooled prevalences of 2.55% for thiuram mix, 0.86% for mercapto mix and 0.83% for mercaptobenzothiazole, with a statistically significant decline in all three after the year 2000, attributed in part to manufacturers substituting dithiocarbamates for thiurams in gloves; thiurams nevertheless remain the single accelerator group with the highest positivity rate, and as many as 40% of patients with rubber-additive allergy are missed if an extended rubber series is not tested alongside the baseline one [35], whereas immediate-type (type I) allergy to natural rubber latex proteins, once a major occupational hazard, has receded since the shift from powdered latex to low-protein and nitrile gloves. The practical implications follow from the mechanism: unnecessary glove use should be avoided, gloves removed periodically and the hands kept dry, cotton liners and accelerator-free nitrile gloves used in selected workers, and emollients maintained as the foundation. Distinguishing irritant occlusion dermatitis from accelerator allergy is not academic—it determines whether the remedy is better glove hygiene or strict avoidance of a specific allergen—and it is one more setting in which the diagnostic algorithm and a low threshold for patch testing earn their place [36].

## 4. Pathophysiology

**Figure 2:**
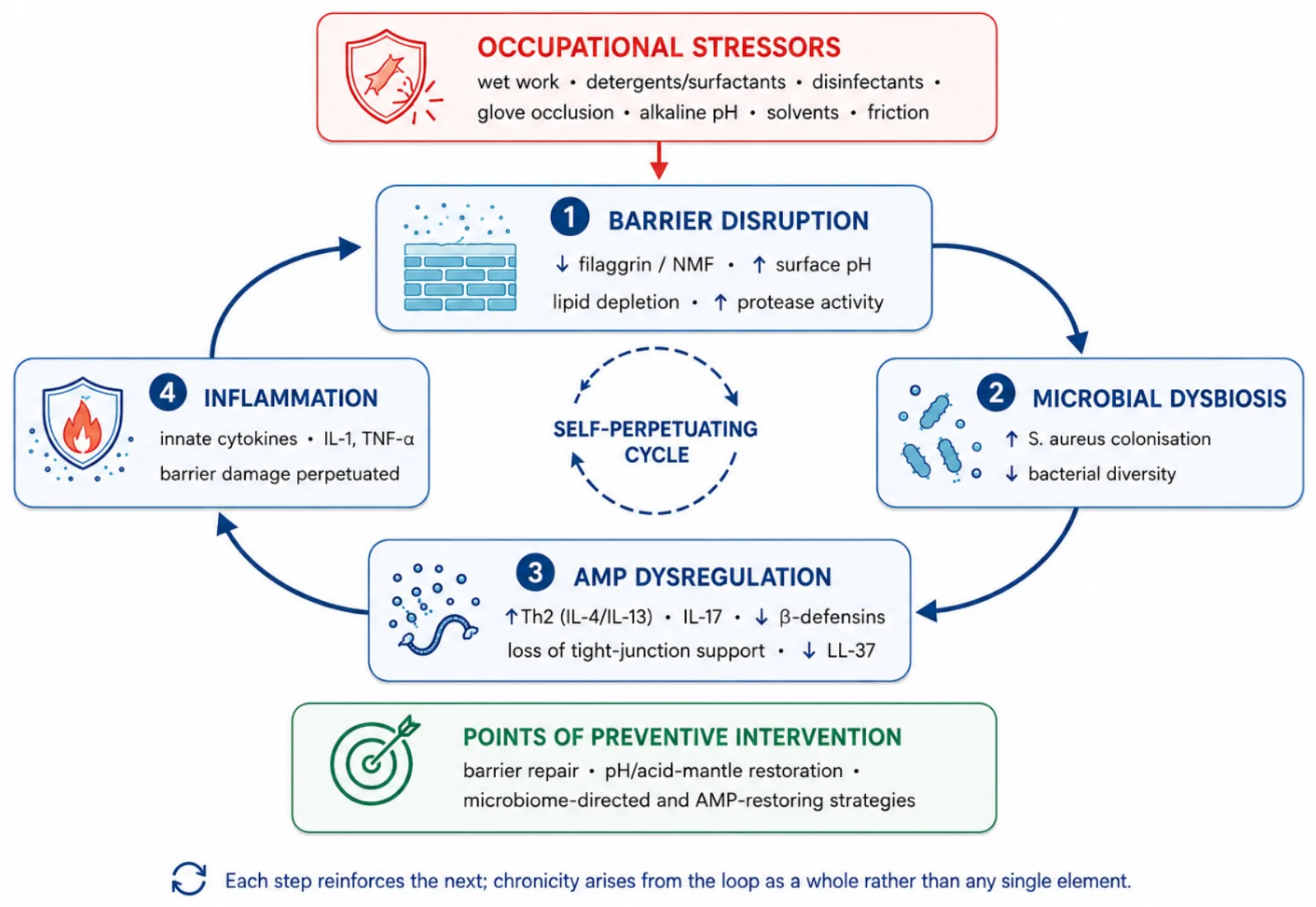
The self-perpetuating cycle of occupational contact dermatitis. Occupational stressors breach the skin barrier; barrier disruption permits microbial dysbiosis (notably *Staphylococcus aureus* overgrowth and loss of diversity); dysbiosis and the Th2-skewed milieu drive antimicrobial-peptide dysregulation, which both removes antimicrobial defence and withdraws the tight-junction support that LL-37 normally provides; the resulting inflammation damages the barrier further. Each step reinforces the next, and the loop as a whole—rather than any single element—underlies chronicity. Barrier repair, acid-mantle restoration, and microbiome- or peptide-directed approaches represent potential points of preventive intervention.

The development of occupational contact dermatitis is best understood not as a single insult but as a self-reinforcing cycle. A breach in the skin barrier alters the microbial community living on it; that altered community engages the innate immune system; the resulting inflammation damages the barrier further. Each of the four elements that follow—barrier, microbiome, innate immunity, and the adaptive response that distinguishes allergic from irritant disease—feeds the next, and it is the loop as a whole, rather than any one component, that explains why occupational hand eczema is so prone to becoming chronic (Figure 2). We take each in turn. One caveat applies to the whole of what follows and is better stated at the outset than buried in qualifications. Much of the mechanistic evidence for the individual links in this cycle has not been generated in occupational hand eczema itself. The barrier and antimicrobial-peptide data derive predominantly from atopic dermatitis; the dysregulated protease-cathelicidin axis is best characterised in rosacea; the immunology of sensitisation rests largely on experimental murine models and human patch-test studies; and several of the microbiological and peptide observations come from chronic wounds or from in vitro keratinocyte systems. We indicate the source population at each step and reserve statements framed as occurring in occupational hand eczema for findings actually obtained in that setting. Elsewhere, the account should be read as a coherent extrapolation awaiting confirmation rather than as established occupational pathophysiology.

### 4.1. Skin Barrier Dysfunction

The stratum corneum is conventionally pictured as a brick-and-mortar wall: anucleate corneocytes embedded in a matrix of intercellular lipid lamellae. The corneocytes are reinforced internally by the cornified envelope—a cross-linked insoluble protein shell—and held to one another by corneodesmosomes. This wall is not inert. It is continuously assembled, processed, and shed, and each of those steps offers a point at which occupational exposures can intervene.

Central to barrier competence is filaggrin, encoded by *FLG* within the epidermal differentiation complex on chromosome 1q21. During terminal differentiation, profilaggrin is processed into monomers that aggregate the keratin cytoskeleton and compact the corneocyte, then degraded into free amino acids and their derivatives—urocanic acid from histidine, pyrrolidone carboxylic acid from glutamine—that together form the natural moisturising factor (NMF). NMF binds water, acidifies the corneum, and even contributes antimicrobial activity, so its loss is felt across several barrier functions at once. Critically for occupational disease, its level is not fixed by genotype alone: stratum-corneum measurements of pyrrolidone carboxylic acid, urocanic acid and histidine tracked both filaggrin genotype and disease severity, indicating that inflammation itself depletes NMF independently of the underlying genetics [37]. Loss-of-function null alleles in *FLG* (most commonly R501X and 2282del4 in European populations) reduce NMF in a dose-dependent fashion and constitute the strongest known genetic risk factor for atopic dermatitis [38].

For occupational disease specifically, the risk attached to *FLG* status has been quantified, and the figures are worth stating precisely. Heterozygous carriage of common null alleles was associated with chronic irritant contact dermatitis at an odds ratio of 1.91 (95% CI 1.02–3.59) [39]. In a German cohort of occupational irritant disease, the crude odds ratio was 2.09 (95% CI 1.33–3.28), attenuating but remaining significant after adjustment for atopic dermatitis (1.62; 95% CI 1.01–2.58)—while atopic dermatitis itself carried an odds ratio of 2.89 (95% CI 2.09–3.99), a reminder that endogenous predisposition and barrier genetics act together [40]. The effect is steepest in heavily exposed trades: among construction workers, *FLG* carriers showed odds ratios of 5.71 (95% CI 1.63–20.06) for mild and 8.26 (95% CI 2.32–29.39) for severe contact dermatitis [41]. One subtlety deserves mention, because it cuts against the grain of these numbers. Individuals who carry *FLG* mutations and developed hand eczema or atopic dermatitis in childhood tend to avoid irritant occupations in the first place [42]. This healthy-worker self-selection can attenuate the measured association in any cross-sectional workforce, and it argues for the value of pre-employment counselling rather than against the relevance of barrier genetics.

The mortar matters as much as the bricks. The intercellular lamellae consist of ceramides, cholesterol, and free fatty acids in roughly equimolar proportion, secreted from lamellar bodies as the cell transits the granular layer. Ceramides—of which there are multiple subclasses defined by sphingoid base and fatty-acid chain length—make up around half the lipid mass, and chain length governs how tightly the lamellae pack. In eczematous and barrier-disrupted skin, there is less total ceramide, a shift toward shorter chains, reduced cholesterol, and looser lipid organisation, all of which track with elevated transepidermal water loss [43]. The therapeutic corollary is that restoring a physiological lipid mixture accelerates barrier repair, which is the rationale behind ceramide-dominant and biomimetic emollients [44].

Two regulatory systems complete the picture. The first is pH: the healthy corneum maintains an acidic surface—the acid mantle—that restrains the kallikrein-related peptidases (KLK5, KLK7, KLK14) responsible for orderly desquamation, their activity held in check at acidic pH by the inhibitor LEKTI, the product of *SPINK5* [45]. The catastrophe of removing that brake is visible in Netherton syndrome, where *SPINK5* loss-of-function unleashes unopposed protease activity and a profound barrier defect [46]. Occupational exposures reproduce a milder version of the same logic: soaps, detergents, occlusion, and alkaline cement all raise stratum-corneum pH and prolong protease activity. The second system is the tight junction—claudin-1, occludin, and the scaffold ZO-1—a second barrier in the granular layer; claudin-1 falls in atopic skin in proportion to Th2 activity, and we will see shortly that one antimicrobial peptide helps hold this junction together, part of why barrier and immunity cannot be cleanly separated [47].

How, concretely, do the exposures of working life damage all this? Surfactants are the prototype. Sodium lauryl sulphate, the reference irritant of experimental dermatology, is small enough to intercalate into the intercellular lipid lamellae and disorder them, denatures and swells corneocyte proteins, and raises stratum-corneum pH. Measured directly under patch-test conditions, it depletes natural moisturising factor and alters corneocyte surface topography; notably, the same changes followed exposure to common contact allergens, which is one more indication that the irritant and allergic routes converge on the same barrier compartment [48]. Wet work acts more mechanically, with repeated swelling and drying cycles disrupting both lipids and corneocytes—the dominant exposure in nursing, hairdressing, food handling, and cleaning. Glove occlusion sustains hydration and elevates pH, impairing repair and amplifying the penetration of any irritant present. Alkaline agents prolong protease activity; organic solvents extract lipid outright; friction abrades the corneum directly. The unifying readout is transepidermal water loss, which rises measurably as disease develops: in a three-year cohort of apprentice nurses, those who reported hand dermatitis at the final examination showed a significant increase at the dorsal hand, from 10.15 to 13.55 g/m2/h. The same cohort is instructive about the limits of the measurement, however. Baseline values recorded before training did not differ between apprentices who later reported symptoms and those who did not, and the authors concluded explicitly that a raised basal transepidermal water loss is not a good indicator of hand dermatitis risk [49]. Transepidermal water loss is therefore best understood as a marker of established barrier damage and as an outcome measure, not as a pre-employment screening test.

### 4.2. The Skin Microbiome and Dysbiosis

Healthy skin carries a characteristic community dominated by *Staphylococcus*, *Corynebacterium*, and *Cutibacterium* species, together with the yeast *Malassezia*, distributed differently across sebaceous, moist, and dry sites. The hands, being dry and continuously exposed, host a comparatively diverse and environmentally responsive flora [50]. In hand eczema, this community shifts in a reproducible direction. *Staphylococcus aureus*, uncommon on healthy hands, colonises a majority of affected ones: in one controlled study it was found on the hands of 54% of patients versus 2% of controls, and across studies its prevalence and density rise with disease severity [51]. Colonisation is not a transient passenger, either—in a prospective cohort, nearly half of patients carried *S. aureus* at two or more visits, most retaining the same clonal type over time, and persistent carriage tracked with worse disease [52].

Alongside this overgrowth comes a measurable loss of diversity. A three-week prospective study with complete follow-up found bacterial alpha-diversity reduced on patients’ hands at baseline, with an effect size of −0.31 (95% CI −0.50 to −0.11; *p* = 0.003), and the dysbiosis was stable rather than fluctuating, correlating with severity [53]. A longitudinal cohort followed over more than six years—and, as it happened, into the COVID-19 pandemic—added a directly occupational dimension: worsening hand eczema was associated with increased *Staphylococcus*, and a high frequency of home handwashing was associated with both lower diversity and higher abundance of *Staphylococcus*, *Corynebacterium*, and other genera, with diversity recovering as time since the last wash increased [54]. The same study found that more frequent moisturiser use shifted community composition, which hints that barrier care and microbial ecology are linked in ways prevention strategies have not yet fully exploited.

The relationship runs in both directions, and the molecular detail makes the loop concrete. Filaggrin’s breakdown products, urocanic acid and pyrrolidone carboxylic acid, directly inhibit *S. aureus* growth, so the same reduced NMF and raised pH that weaken the barrier also remove a chemical brake on staphylococcal overgrowth [55]. The bacterium returns the favour: its proteases and toxins degrade barrier proteins and amplify inflammatory and Th2 signalling. That a fortnight of topical corticosteroid not only calms inflammation but also increases microbial diversity and reduces *S. aureus*, shifting the lesional community back toward its non-lesional state, shows that the dysbiosis is at least partly reversible and is tied to the inflammatory state rather than fixed [56].

### 4.3. Innate Immunity and Antimicrobial Peptides

Keratinocytes are not passive bystanders to all this; they are immune sentinels. They express pattern-recognition receptors—several Toll-like receptors and cytosolic NOD-like receptors—and assemble the NLRP3 inflammasome, which on sensing danger activates caspase-1 to cleave pro-IL-1β and pro-IL-18 into their active forms [57]. The triggers for this machinery are precisely the damage signals that irritants generate: extracellular ATP, fragments of hyaluronic acid, and reactive oxygen species. The role of ATP is illustrated cleanly in animal models of contact hypersensitivity, where signalling through the P2x7 receptor is required to mount a response—its genetic absence, or pharmacological blockade, prevents sensitisation [58]. The IL-1 family that sits at the centre of this response is shared by both forms of contact dermatitis, which is one reason the two are mechanistically harder to separate than their clinical labels suggest.

The antimicrobial peptides deserve particular attention, both because they are the chemical interface between barrier and microbiome and because they connect to ongoing work on peptide therapeutics and smart biomaterials. Cathelicidin, processed from its precursor into the active peptide LL-37, is induced by vitamin D signalling, by barrier disruption, by wounding, and by infection. It is directly antimicrobial, but it does more than kill: LL-37 upregulates the tight-junction proteins claudin and occludin and increases the electrical resistance of keratinocyte layers, so that a peptide we think of as antimicrobial is simultaneously a barrier-reinforcing molecule [59]. The β-defensins (hBD-1 constitutively, hBD-2 and hBD-3 on induction) add broad-spectrum activity, hBD-3 being especially potent against *S. aureus*; dermcidin from sweat, RNase 7, and psoriasin round out the repertoire [60].

Here the loop closes on itself. Cathelicidin and the inducible β-defensin hBD-2 are expressed at significantly lower levels in atopic than in psoriatic lesions, and the two peptides act synergistically against *S. aureus*, so that their relative deficiency plausibly underlies the staphylococcal susceptibility of eczematous skin [61]; the Th2 cytokine milieu itself, and IL-4 and IL-13 in particular, prevents the induction of these innate immune response genes [62]. The consequence is a double hit: less direct antimicrobial defence, permitting the *S. aureus* overgrowth already described, and less of the tight-junction support that LL-37 normally provides, weakening the barrier further. A breached barrier favours dysbiosis; dysbiosis and the Th2 skew suppress the peptides that would defend the barrier; their loss deepens both the microbial and the structural defect. This barrier–dysbiosis–antimicrobial-peptide–inflammation circuit is, in our view, the single most useful organising idea for the pathophysiology of occupational dermatitis, precisely because it identifies several points—barrier repair, pH restoration, and peptide- or microbiome-directed approaches—at which prevention might intervene. The principal cutaneous antimicrobial peptides, their cellular sources, and their relevance to occupational disease are summarised in Table 3.

### 4.4. A Conceptual “Trans-Kingdom Dialogue” Model Linking Host Antimicrobial Peptides and Staphylococcal Quorum Sensing

**Figure 3:**
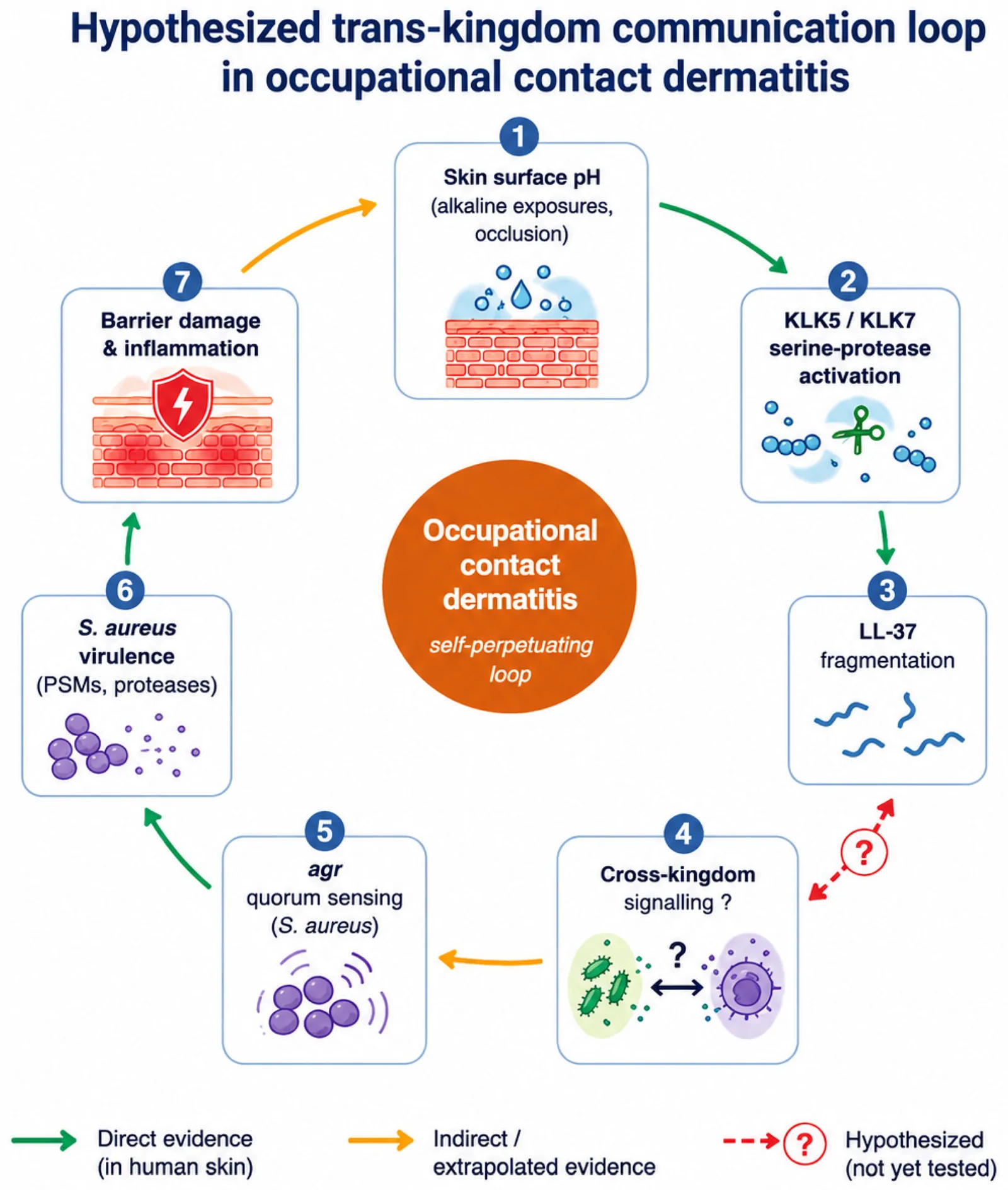
Hypothesised trans-kingdom communication loop in occupational contact dermatitis. The occupational microenvironment—alkaline pH and glove occlusion—activates stratum-corneum kallikreins, which fragment the cathelicidin LL-37; we hypothesise that these fragments may act as cross-kingdom signals modulating the *Staphylococcus aureus agr* quorum-sensing system, promoting virulence, barrier damage, and the further rise in pH that sustains the cycle. Each arrow is coloured by the strength of the supporting evidence: green, direct evidence in human skin; amber, indirect or extrapolated evidence; red (dashed), hypothesised and not yet tested. The step from LL-37 fragments to *agr* signalling (marked ‘?’) is the principal untested conjecture. *agr*, accessory gene regulator; KLK, kallikrein-related peptidase; PSM, phenol-soluble modulin.

The preceding subsections follow a logical sequence—a disrupted barrier (Section 4.1) permits microbial dysbiosis (Section 4.2), which unfolds against a background of dysregulated antimicrobial-peptide defence (Section 4.3). Here we propose that there may be one further layer to this account. Beyond their established roles as structural and antimicrobial elements, host antimicrobial peptides and the bacteria they target may be engaged in active molecular cross-talk, such that occupational contact dermatitis is perhaps best understood not solely as a disease of barrier failure but, in part, as a disorder of communication between host and microbe. To our knowledge, this conceptual framework has not previously been proposed in occupational contact dermatitis; related host–microbe ideas have been advanced in atopic dermatitis [63] and rosacea [64], but not as an integrated, occupationally driven loop (Figure 3). We present the model deliberately as a hypothesis, and for each step we indicate whether the supporting evidence is direct in human skin, indirect or extrapolated from other organisms and diseases, or as yet untested.

The proposed loop begins with the workplace itself. Repeated contact with alkaline detergents and wet cement, together with the sweat and occlusion beneath gloves, raises stratum-corneum pH [65]; an alkaline surface impairs barrier repair, favours *Staphylococcus aureus*, and—most relevant here—sustains the activity of the stratum-corneum serine proteases, the kallikrein-related peptidases KLK5, KLK7, and KLK14. That unopposed kallikrein activity is by itself sufficient to cause severe disease is shown by Netherton syndrome, in which loss of their inhibitor LEKTI removes the brake entirely [45,46]. This protease-rich environment is reinforced by the microbes themselves, since *S. aureus* induces keratinocyte serine-protease activity and so accelerates barrier breakdown [66]. Crucially, the same proteases that degrade barrier proteins also cleave cathelicidin, and the principle that protease-generated cathelicidin fragments are not inert by-products but can be actively pro-inflammatory is well established in human skin: in rosacea, abnormal KLK5-mediated processing of LL-37 generates peptide fragments that drive inflammation [64]. We therefore take as our starting point a reasonably well-supported premise—that the occupational microenvironment fosters proteolytic fragmentation of LL-37.

The more speculative element of the model concerns what those fragments may then do to the microbial community. We hypothesise that, beyond any change in their antimicrobial potency, LL-37 and its proteolytic fragments may act as cross-kingdom signalling molecules that influence staphylococcal behaviour—in particular the accessory gene regulator (*agr*) quorum-sensing system through which *S. aureus* coordinates virulence. Direct proof that a host cathelicidin fragment can serve as a bacterial signal does exist, although in a different organism: in group A *Streptococcus*, LL-37 activates virulence-gene expression through the CsrRS two-component system [67] and binds directly to the sensor histidine kinase CsrS, while a defined ten-residue fragment (RI-10) retains this signalling activity while having no detectable antimicrobial activity at all [68]—a clean demonstration that the two functions can be uncoupled. For *S. aureus,* the evidence is thinner and only indirect: sub-inhibitory LL-37 has been reported to alter expression of *agr* and its effector *RNAIII* in biofilm-forming isolates from chronic wound infections, although the statistically significant finding in that study was a difference in response between methicillin-resistant and methicillin-susceptible strains rather than a uniform effect [69]. Whether defined LL-37 fragments modulate the *S. aureus agr* system in skin has, to our knowledge, never been tested. It is conceivable that, in the alkaline and protease-rich niche of the occupationally damaged hand, the particular repertoire of LL-37 fragments generated could bias *S. aureus* toward a more virulent, *agr*-active state; we emphasise, however, that this specific step remains unproven and is offered as a hypothesis to be tested rather than a mechanism to be assumed.

If such signalling does occur, its downstream consequences are easier to anchor in evidence. Through *agr*, *S. aureus* controls the phenol-soluble modulins and secreted proteases that themselves damage the epithelial barrier—a relationship demonstrated directly in atopic dermatitis, where commensal coagulase-negative staphylococci that quorum-quench *agr* limit this injury [63]. The loop then closes where it began: *agr*-driven damage and the *S. aureus*-dominated, low-diversity community repeatedly documented on eczematous hands [51,52,53,56] perpetuate the inflammation and protease activity that generated the fragments in the first place, while sustaining the elevated pH that keeps the cycle turning. Viewed this way, chronic occupational hand eczema comes to resemble, at the molecular level, the dysregulated antimicrobial-peptide and protease environment of the chronic wound, raising the possibility that mechanistic insight may be transferable between the two. Two tensions must be stated plainly rather than glossed over. First, the *agr* system inversely regulates biofilm formation, so *agr* inhibition can paradoxically promote biofilm; the model cannot assume that quenching *agr* is uniformly beneficial. Second, the best-characterised example of staphylococcal processing of the peptide points the other way: staphopain B cleaves LL-37 into shorter fragments, and while the intact peptide significantly inhibited biofilm formation, the degradation products did not [70]. Our hypothesis therefore requires the more specific proposition that signalling activity may be retained or even acquired as direct antimicrobial activity is lost—for which the streptococcal fragment data [68] remain the clearest precedent.

Framed as a disorder of host–microbe communication, the model recasts prevention and treatment as opportunities to interrupt a dialogue rather than merely to suppress inflammation. The most conservative and immediately testable lever is restoration of an acidic surface pH and barrier integrity, which would be expected to dampen kallikrein activity, limit fragment generation, and disfavour *S. aureus*. More ambitious—and at present unproven in skin—are approaches that target the bacterial conversation directly, whether by quorum quenching or by microbiome-directed therapy; the real limitations of quorum-sensing-modulating strategies, together with the broader precision-medicine context, are considered in Section 8 [71].

The value of a conceptual model lies in whether it can be falsified, and this one yields four predictions that are achievable with existing biomarker, mass-spectrometric and sequencing tools. Skin-surface pH, kallikrein activity and disease severity should covary on affected hands and exceed the values measured on the unaffected hands of the same worker; the stratum-corneum LL-37 fragment repertoire should differ in occupational hand eczema from that of healthy but comparably exposed skin; the *agr* activity of colonising staphylococci should correlate with that fragment profile and with clinical severity; and defined LL-37 fragments applied to *S. aureus* in vitro at sub-inhibitory concentrations should measurably alter *agr* and *RNAIII* expression, which is the single most direct test of the central conjecture. Section 8 considers how such measurements might be embedded in prospective occupational cohorts, and we note only that interventions re-acidifying the skin should reduce both fragment burden and *agr*-driven virulence markers before any effect on visible inflammation becomes apparent.

### 4.5. The Immunology of Irritant Versus Allergic Contact Dermatitis

With this shared innate machinery in mind, the distinction between the two forms of contact dermatitis can be drawn accurately. Irritant contact dermatitis is fundamentally an innate event. The irritant damages keratinocytes directly; the damaged cells release preformed IL-1α, and the danger signals that activate the inflammasome and NF-κB; and the result is a cascade of innate cytokines—IL-1α and IL-1β, IL-6, TNF-α, the neutrophil chemokine CXCL8, IL-18, IL-23—amplified by neurogenic inflammation as sensory nerves release substance P and CGRP [72]. There is no antigen-specific memory and no requirement for prior exposure; the response is governed by dose and by the threshold of the individual barrier, which is exactly why filaggrin status and the other barrier factors weigh so heavily on susceptibility.

Allergic contact dermatitis adds a layer that irritant disease lacks: a hapten-specific adaptive response, the textbook example of delayed-type (type IV) hypersensitivity. Its mechanism unfolds in two phases. During sensitisation, small reactive haptens penetrate the corneum and covalently modify epidermal proteins; the same cellular stress that irritants cause provides an obligatory innate danger signal; and dendritic cells, having captured the hapten-protein conjugate, mature and migrate to the draining lymph node, where they prime naive hapten-specific T cells [73]. On re-exposure—the elicitation phase—skin-homing effector T cells drive the visible reaction. The principal effectors are CD8+ cytotoxic T cells, of both the IFN-γ-producing and IL-17-producing types, regulated by CD4+ cells, and their killing of keratinocytes proceeds through the Fas and perforin/granzyme pathways, which are functionally redundant such that either suffices [74,75]. The reaction is eventually contained by regulatory T cells, which act at the skin surface through IL-10 and TGF-β and, in murine contact hypersensitivity, through CD39-generated adenosine that blocks leukocyte adhesion to the endothelium [76].

The most important conceptual point is that these two processes are not truly separate. Sensitisation to a contact allergen absolutely requires innate activation—the very machinery that drives irritant dermatitis—and irritants accordingly act as adjuvants, lowering the threshold for and enhancing sensitisation. In the workplace this is the rule rather than the exception: occupational hand eczema is frequently a mixed picture, an allergic response superimposed on irritant damage on a constitutionally barrier-defective background. Recent molecular work bears this out while also offering a way to tell the two apart. Single-cell transcriptomic comparison of experimentally induced allergic and irritant reactions found that the distinction lies less in which cells are present than in what those cells are transcribing, with allergic reactions marked by an expansion of CD8+ T cells and cytotoxic natural killer cells and a clear interferon-γ signature, including keratinocyte production of the chemokines CXCL9, CXCL10, and CXCL11 [77]. Focused gene panels built around cytotoxic and type-1 markers can now discriminate allergen- from irritant-induced reactions [78], and in one series of 38 patients with active contact dermatitis a 12-biomarker panel separated the cohort into an allergy-signature group and an irritant-signature group: the allergy signature contained all 17 patients whose allergic disease had been confirmed by patch testing and exposure assessment, together with six further patients in whom no culprit allergen had been identified, while every patient without biomarker induction had a negative patch test [79]. The clinical promise therefore lies less in overturning positive patch tests than in identifying an allergic profile when patch testing has failed to reveal the responsible allergen. These signatures remain to be validated in chronic clinical hand eczema rather than experimental patch-test settings, but they point toward a future in which the irritant-versus-allergic question is settled molecularly rather than inferred.

Finally, the specific allergens of working life are worth grounding in chemistry, because their mechanisms are not interchangeable. Nickel, the most frequent contact allergen on patch testing, is exceptional in activating human innate immunity directly, binding TLR4 at histidine residues present in the human but not the murine receptor—hence the natural resistance of mice to nickel sensitisation [80]. Chromium enters cells as chromate and is reduced intracellularly to its protein-reactive form, the hexavalent species supplying the danger signal through reactive oxygen and inflammasome activation [81]. p-Phenylenediamine, the hairdresser’s allergen, is a prohapten requiring oxidation before it can modify protein [82]; isothiazolinone preservatives react with cysteine thiols [83]; epoxy resins open their reactive ring onto protein nucleophiles [84]; and the thiuram and dithiocarbamate accelerators behind glove allergy form mixed disulfides with protein thiols, with thiuram mix still the leading rubber-accelerator allergen on patch testing [35]. The common thread is electrophilic or redox reactivity toward protein—the basis of haptenation—but the route differs enough between allergens to shape both the clinical pattern and the occupations at risk.

## 5. Diagnosis

**Figure 4:**
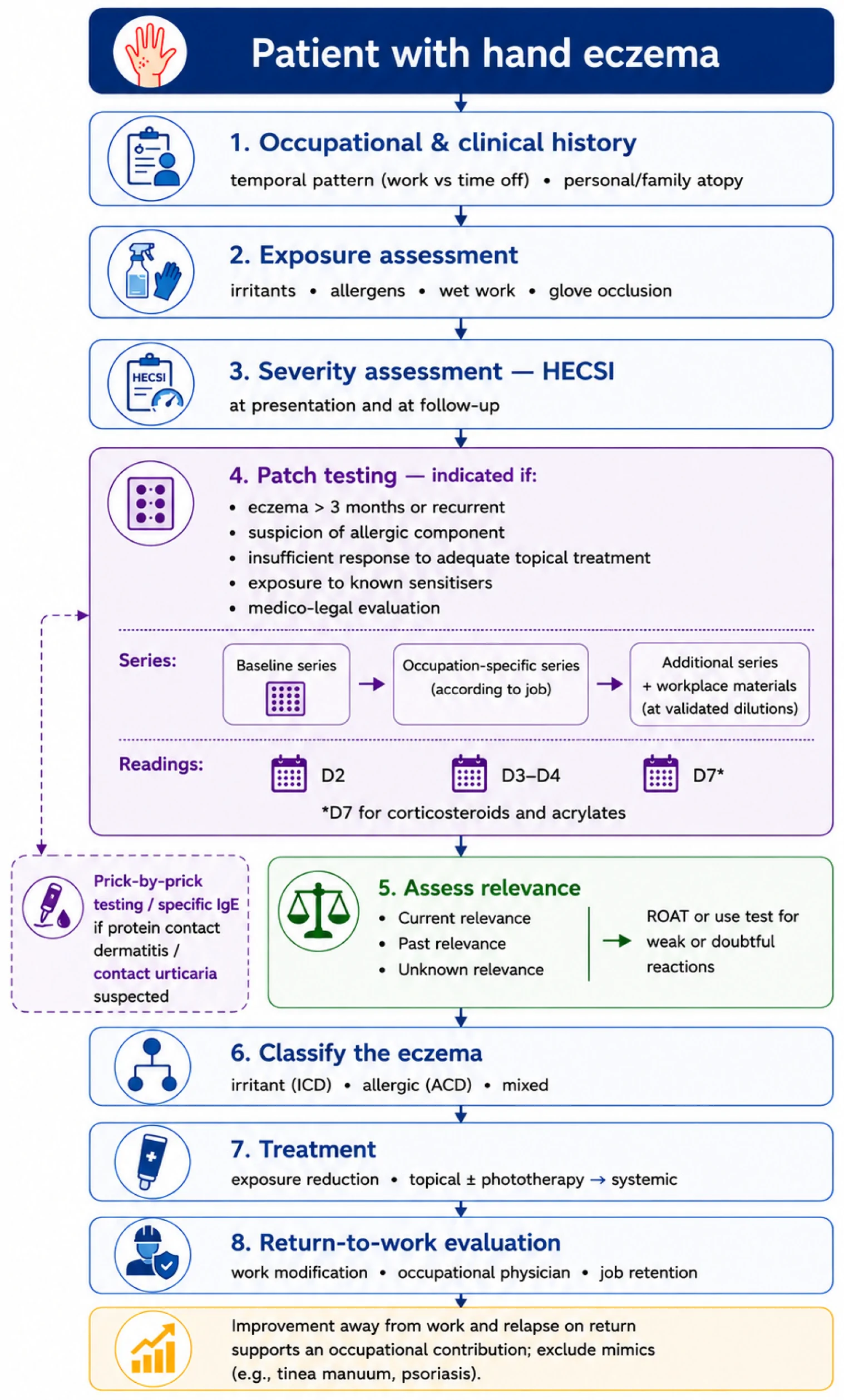
A practical diagnostic algorithm for occupational hand eczema. Assessment proceeds from a structured occupational and clinical history, with attention to the temporal relationship between symptoms and work, through exposure assessment to severity scoring with the Hand Eczema Severity Index at presentation and at follow-up. Patch testing is shown as a separate decision step rather than as a routine measurement running in parallel with severity scoring: the clinical circumstances that prompt it are listed, and testing proceeds from the baseline series to an occupation-specific series and, where the relevant allergen is not covered by these panels, to additional series and the patient’s own workplace materials at validated dilutions, with readings at day 2 and day 3 to 4 and a delayed reading at day 7 for corticosteroids and acrylates. Because a positive reaction is not in itself diagnostic, relevance is assessed as a distinct step and recorded as current, past or unknown, with repeated open application or use testing reserved for weak or doubtful reactions. Where protein contact dermatitis or contact urticaria is suspected, immediate-type testing by prick-by-prick and specific IgE runs as a parallel branch. Classification as irritant, allergic or mixed disease then informs treatment and a formal return-to-work evaluation. Improvement away from work with relapse on return supports an occupational contribution; clinical mimics such as tinea manuum and psoriasis should be excluded. HECSI, Hand Eczema Severity Index; ICD, irritant contact dermatitis; ACD, allergic contact dermatitis; ROAT, repeated open application test; IgE, immunoglobulin E.

Diagnosing occupational contact dermatitis is, in practice, a sequence of linked judgements rather than a single test: establishing that hand eczema is present and grading its severity, determining whether an allergic component is involved, and deciding whether the condition is genuinely work-related. None of these steps is wholly straightforward, and the difficulty of the second in particular—telling allergic, irritant, and endogenous disease apart—runs through everything that follows. Figure 4 sets out this diagnostic sequence as a practical algorithm.

A reliable diagnosis begins before any test, with the history. Three questions do most of the work: what the hands are exposed to across a full working day, how the eruption behaves in relation to time away from work, and whether there is a personal or family background of atopy. The temporal pattern is especially informative. Disease that eases during weekends or holidays and flares on return points toward an occupational contribution, whereas an eruption indifferent to time away from the workplace argues for an endogenous process—though, as ever, the two can coexist, and improvement on leave does not by itself distinguish irritant from allergic causation.

Severity is best captured with a validated instrument, and the Hand Eczema Severity Index (HECSI) has become the reference. It scores six morphological signs—erythema, infiltration and papulation, vesicles, fissures, scaling, and oedema—across five regions of the hand, yielding a total that ranges from 0 to 360 [85]. Validated severity bands give the raw number clinical meaning: a score of 0 corresponds to clear skin, 1 to 16 to almost clear, 17 to 37 to moderate, 38 to 116 to severe, and 117 or above to very severe disease [85]. The instrument’s inter- and intra-observer reliability is high, which makes it suitable not only for clinical follow-up but for the serial measurement that prevention trials and return-to-work assessments depend on—indeed, several of the studies discussed elsewhere in this review use HECSI change as their primary endpoint.

Morphology and distribution add a further layer, even if they rarely settle the question alone. Irritant disease tends to begin in the finger webs and on the dorsa of the hands, the sites of greatest exposure and thinnest barrier, and to track closely with the intensity of wet work or friction. Allergic disease often takes the pattern of the contactant—the fingertips in those handling small allergenic objects, the dorsal hand and wrist where gloves sit—and may extend beyond the area of obvious contact. Several morphological subtypes are worth naming because they shape both the differential and the prognosis: a chronic fissured hyperkeratotic pattern of the palms, a recurrent vesicular (pompholyx-like) pattern, and a nummular pattern each carry different associations and different responses to treatment. None of these is pathognomonic, and the value of describing them lies less in clinching a cause than in framing the differential and tracking change over time.

That differential is broad, and missing an alternative diagnosis is a common pitfall. Palmar psoriasis can closely mimic chronic hyperkeratotic hand eczema and may need the rest of the skin, the nails, and occasionally a biopsy to distinguish. Tinea manuum—classically affecting one hand and both feet—must be excluded by mycology before a presumed eczema is treated with topical corticosteroids, which would otherwise mask and prolong it. Scabies, palmoplantar pustulosis, and, in atypical or treatment-resistant cases, mycosis fungoides all enter the differential. The practical rule is that hand eczema failing to respond as expected deserves reconsideration rather than escalation, and a low threshold for mycological sampling and, where doubt persists, biopsy.

The cornerstone of the allergic work-up is patch testing. A baseline series—in Europe, the European baseline series—is applied to identify the common contact allergens, and supplementary occupational series are added according to the trade in question: hairdressing, metalworking, and rubber series each capture allergens that the baseline panel would miss. Patch testing is what converts a clinical suspicion of an allergic component into evidence, and without it the irritant-versus-allergic distinction remains an inference. Two further forms of testing connect that sensitisation to the patient’s actual exposures. The repeated open application test, in which a suspected product is applied to the forearm twice daily over one to two weeks, helps determine whether a weakly positive or doubtful patch-test reaction is clinically relevant to a leave-on or rinse-off product the patient genuinely uses. Testing with the patient’s own workplace materials, appropriately diluted and interpreted with care to avoid irritant false positives, can be decisive when a relevant occupational allergen is not represented in the standard series. Both procedures demand caution in the occupational setting, and the repeated open application test in particular should not be described as a straightforward extension of patch testing. Workplace materials are frequently irritant in the form in which they are supplied and must be tested at validated, substance-specific dilutions in appropriate vehicles, with irritant controls and, wherever possible, guidance from published occupational series; testing an undiluted product risks an irritant reaction that will be misread as sensitisation. Even a correctly performed repeated open application test may be confounded on skin that is already inflamed, and where a suitable dilution or vehicle has not been established, referral to a specialised occupational patch-test centre is preferable to improvised in-house testing. Where a protein contact dermatitis or contact urticaria is suspected—in food handlers and healthcare workers exposed to natural rubber latex, for instance—the relevant investigations shift toward immediate-type testing, with skin prick testing and specific IgE rather than delayed-reading patches, since the underlying mechanism is different and a delayed-reading patch test would not capture it.

Non-invasive biophysical measurement and skin imaging are a useful adjunct where the clinical and patch-test picture remains ambiguous, although neither currently replaces patch testing. Transepidermal water loss quantifies barrier integrity and rises measurably as barrier damage develops, although, as noted in Section 4.1, baseline values do not predict who will go on to develop hand eczema [49]; in experimentally induced reactions the increase is significantly greater for irritant than for allergic responses, which gives it a modest discriminant value alongside its role as an early-detection and outcome measure. In vivo reflectance confocal microscopy resolves spongiosis, intra-epidermal vesiculation and inflammatory-cell traffic in real time, and in comparative studies of induced reactions the irritant lesions showed greater disruption of the stratum corneum and more parakeratosis than the allergic ones [86]. Optical coherence tomography, including its high-definition and line-field confocal variants, permits non-invasive measurement of epidermal thickness and has been proposed both as an aid to patch-test grading and as a means of separating doubtful positive from irritant reactions [87,88]. For occupational hand eczema specifically, these methods remain research tools rather than clinical ones: the published series are small, the palm and dorsum of the hand are technically demanding sites because of stratum-corneum thickness and surface relief, and prospective validation against patch-test outcome in working populations has not yet been carried out.

Taken together, these tools turn the diagnostic sequence into something more than pattern recognition. The history generates hypotheses, morphology and distribution refine them, the differential guards against mimics, and the testing—delayed-type for allergens, use-oriented for relevance, immediate-type where the mechanism demands it—converts suspicion into evidence. The most current European guidance, the German S2k guideline on the diagnosis, prevention, and therapy of hand eczema, sets out a stepwise approach organised by severity and provides the practical framework most clinicians will recognise [36]. Referral to occupational dermatology is warranted when disease is chronic, severe, or recalcitrant to first-line measures, and whenever a formal assessment of work-relatedness is needed for medicolegal or compensation purposes; in many jurisdictions, recognition of an occupational skin disease is what unlocks access to the structured prevention programmes examined in the next section.

## 6. Prevention

Of all the topics in this review, prevention is where the distance between what we understand mechanistically and what we can demonstrate empirically is greatest. The biology set out above points clearly toward barrier protection, pH restoration, and reduced wet work as rational targets. Yet when those rational measures are put to the test of a randomised trial, the results have been sobering. It is worth stating the central tension at the outset: the two methodologically strongest trials of primary and secondary prevention have been essentially negative on their primary outcomes, while the structured rehabilitation programmes that report the most impressive results rest on uncontrolled cohort data. Reconciling these two facts is the task of this section, and it shapes how confidently any preventive recommendation can be made.

### 6.1. The Hierarchy of Controls

**Figure 5:**
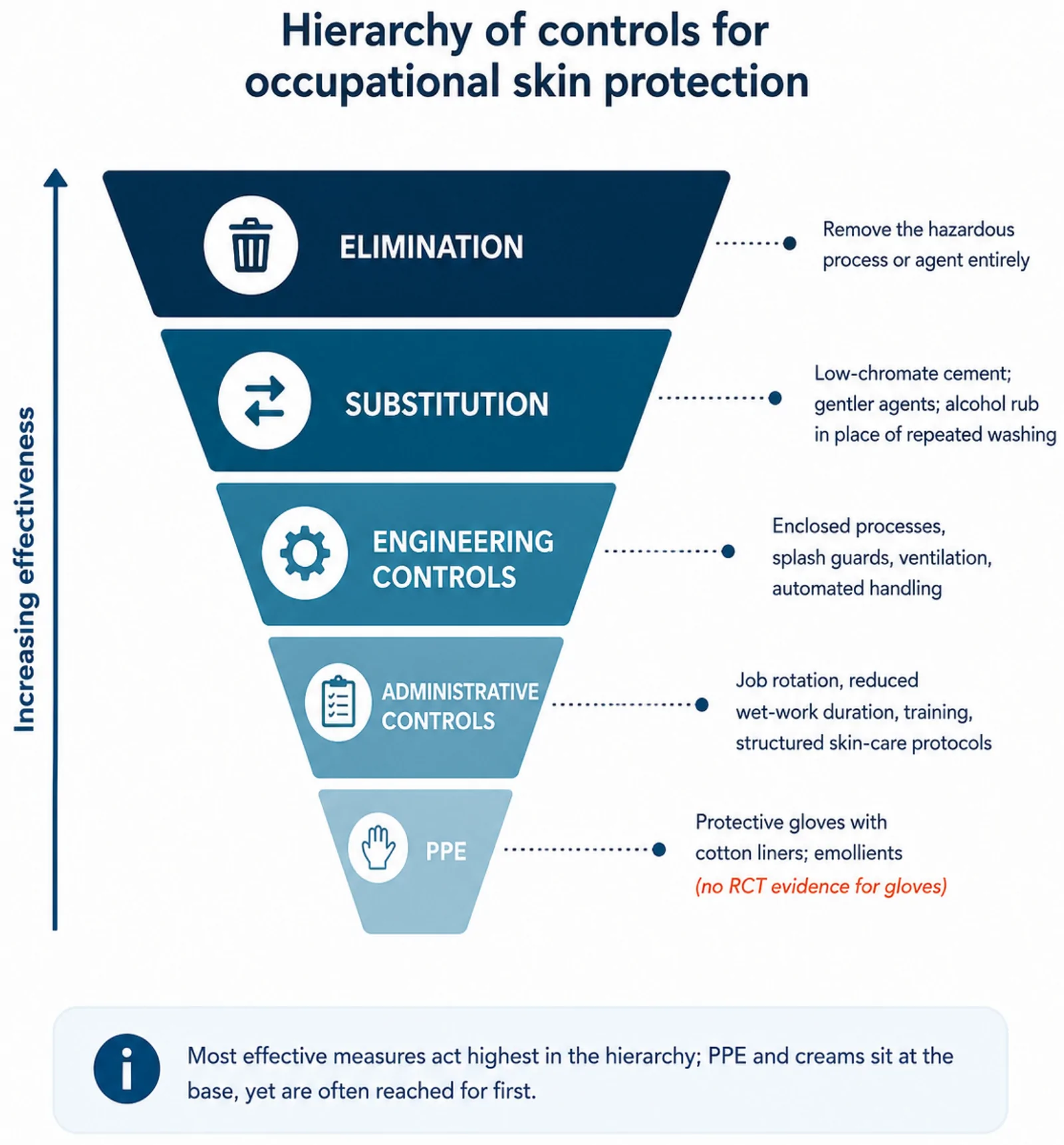
Hierarchy of controls applied to occupational skin protection. Measures highest in the hierarchy—eliminating or substituting the hazard, and engineering controls—are the most effective; administrative measures and personal protective equipment sit lower. In practice, gloves and creams (the base of the hierarchy) are often reached for first, while the more effective upper tiers are left untouched.

The logical framework for occupational skin protection is the standard hierarchy of controls familiar from occupational hygiene, ordered from most to least effective: eliminating the hazard, substituting a less harmful agent, engineering controls that separate worker from exposure, administrative measures such as job rotation and reduced wet-work duration, and—last, not first—personal protective equipment (Figure 5). The ordering matters because the instinct in practice is often to reach straight for gloves and creams, the bottom of the hierarchy, while leaving the more effective upper tiers untouched. Substitution, by contrast, has produced one of the field’s clearest successes. The addition of ferrous sulphate to cement, which reduces hexavalent chromium to its far less sensitising trivalent form, was made mandatory across the European Union by Directive 2003/53/EC with effect from January 2005. In the United Kingdom the incidence of allergic contact dermatitis attributed to chromate fell sharply thereafter: the incidence rate ratio between 2002 and 2004 and 2005–2009 was 0.48 (95% CI 0.36–0.64), against 0.76 (0.69–0.85) for allergic contact dermatitis not attributed to chromate, a significantly greater decline; among workers likely to be exposed to cement the ratio fell to 0.37, and most of the change occurred during 2005 itself [89]. A later analysis extending the assessment to France reached the same conclusion in both countries [90]. This is an elimination-by-substitution intervention achieving what no cream could.

The post-pandemic surge in hand hygiene offers an instructive worked example of the upper tiers. A systematic review and meta-analysis of 45 studies found that handwashing eight to ten or more times a day significantly increased the risk of hand eczema (relative risk 1.51; 95% CI 1.35–1.68), rising to 1.66 (1.51–1.83) at fifteen to twenty or more times a day, while wet work carried a relative risk of 1.37 (1.24–1.51) and alcohol-based hand rub showed no significant association. The certainty of evidence was graded low for the handwashing and wet-work outcomes and very low for hand rub, all of it resting on observational studies [9]. This finding has a direct administrative implication that runs counter to intuition: where hand decontamination is required on intact skin, alcohol-based rub is gentler to the barrier than repeated washing, and prevention messaging should say so rather than leaving workers to assume that more washing is safer. A pragmatic quality-improvement programme during the pandemic, in a single tertiary hospital and involving only 21 healthcare workers, suggested a combined effect of several such measures—substituting a gentler rub, alternating rub with washing, modifying duties temporarily, and switching from latex to nitrile gloves—in improving recovery of irritant hand dermatitis among healthcare workers [91]. None of these is glamorous, and none depends on a novel product; their value lies in addressing exposure rather than merely buffering its consequences.

### 6.2. Primary Prevention and the Limits of the Evidence

The reference point for primary prevention is the 2018 Cochrane review, and its conclusions must be reported faithfully rather than softened. Drawing on nine randomised trials enrolling 2888 participants without irritant hand dermatitis at baseline, the review concluded that barrier creams, moisturisers, and their combination may reduce the development of occupational irritant hand dermatitis, but that the evidence supporting this is of low quality by GRADE criteria [15]. Two further conclusions are as important as the first. There were no randomised trials of protective gloves at all—a remarkable gap given how universally gloves are recommended—and the effect of skin-protection education was judged uncertain [15]. The authors were explicit that this does not mean the measures are ineffective, only that the evidence is insufficient to confirm that they work; the distinction matters, but it is not reassurance.

The two largest trials published since have, if anything, widened rather than closed this gap. The Healthy Hands Project, a single-centre cluster randomised trial across nineteen hospital wards and some five hundred nurses, equipped intervention wards with cream dispensers, electronic monitoring, and feedback. The change in hand eczema severity favoured the intervention numerically—a HECSI reduction of 6.2 against 4.2 in control wards—but the difference did not reach significance on the primary outcome, achieving significance only within the subgroup of nurses with mild disease [16]. The PREVEX trial was larger still, individually randomising 756 workers with newly notified occupational hand eczema to a group educational programme or usual care. All three of its co-primary outcomes—sickness absence, quality of life, and severity—were null, and a post hoc analysis raised the uncomfortable possibility that the intervention was actually detrimental among healthcare workers, with a significant subgroup interaction [17]. Taken together, the strongest evidence available indicates that we do not yet know how to prevent incident occupational hand dermatitis through skin care and education, and that well-intentioned programmes cannot be assumed to help simply because their components are sensible.

### 6.3. Secondary and Tertiary Prevention

The picture changes—though its evidentiary foundation does not strengthen proportionately—when attention turns from preventing disease in healthy workers to managing those already affected and at risk of losing their livelihoods. Germany’s structured, tiered prevention programmes are the most developed model. In the ROQ multicentre cohort, in which 1788 patients with severe occupational skin disease threatened with job loss were enrolled, and 1410 were available for the three-year analysis, an inpatient multidisciplinary rehabilitation programme was followed by striking outcomes at three years: nearly all patients were able to resume work, and 82.7% remained employed, with sustained reductions in severity and absence [21]. A systematic review of German secondary and tertiary prevention, covering 19 studies and 5527 patients, found broadly consistent job retention: approximately 70% to 90% of patients remained in their occupation one year after the secondary programme and 60% to 70% at five years, while 82.7% remained in work three years after the tertiary programme, alongside significant reductions in severity and gains in quality of life [22]. The difficulty is methodological rather than substantive: these are uncontrolled cohorts, heterogeneous in design, and the systematic review explicitly declined to assign a formal certainty grade because the underlying studies could not support one [22].

How, then, should the apparent contradiction be read—null primary-prevention trials alongside strongly positive tertiary-prevention cohorts? The most defensible interpretation is that they are answering different questions in populations of different severity. Intensive, multidisciplinary rehabilitation of established severe disease, delivered to highly motivated workers facing job loss, plausibly produces real benefit, and the consistency of the retention figures across programmes lends it credibility even without randomisation. What is lacking is not evidence that managing severe established disease helps, but rigorous evidence that light-touch primary-prevention programmes reduce the incidence of new disease in the general workforce. The two should not be conflated, and a recommendation appropriate to one tier cannot be borrowed for another.

### 6.4. Barrier-Repair Formulations

Sitting across these tiers is the question of what, specifically, an emollient or barrier product should contain—a question the prevention trials have largely treated as secondary to the act of application itself. A terminological distinction is required first, because the prevention literature has often treated four different classes of product as though they were interchangeable. Barrier creams, also called pre-work creams, are applied before exposure and are intended to reduce penetration of a specific irritant. Emollients and moisturisers are applied after exposure or after the working day and are intended to restore hydration and lipid content, the two terms differing chiefly in regional usage rather than in composition. Skin-protection products are the broader occupational-hygiene and regulatory category encompassing both of these together with mild cleansers and protective gloves. The distinction is not pedantic: the Cochrane analysis pooled barrier creams with moisturisers, and pre-exposure shielding and post-exposure repair are mechanistically different interventions that should not be expected to produce identical effects or to be interchangeable in a preventive regimen. The mechanistic argument developed earlier suggests this is a missed opportunity. Since the barrier defect in occupational and atopic-predisposed skin involves reduced and shortened-chain ceramides, looser lipid organisation, and a disturbed protease–pH balance, formulations designed to replenish the physiological lipid mixture—ceramide-dominant and biomimetic lamellar-lipid emollients [44], and acidic preparations that support the protease-restraining acid mantle [92]—are mechanistically rational in a way that simple occlusive moisturisers are not. Restoring a physiological ratio of ceramides, cholesterol, and free fatty acids accelerates barrier recovery and reduces irritant susceptibility in experimental and atopic settings [44]. The honest caveat is that high-certainty randomised trials testing modern biomimetic emollients specifically for occupational prevention have not yet been done; the rationale is strong, the confirmatory evidence is pending, and this is precisely the kind of question the field most needs answered. The evidence on prevention is summarised in Table 4.

**Table 5:** Principal therapeutic options for chronic and occupational hand eczema, with mechanism, strength of evidence, and regulatory status. Evidence is graded on the four-level qualitative scale defined in the caption to Table 4; “approved” and “off-label” refer specifically to the hand-eczema indication.

Therapy	Mechanism	Evidence	Status (Hand Eczema)
Topical corticosteroids	Broad anti-inflammatory	High	First-line/standard of care
Topical calcineurin inhibitors (tacrolimus, pimecrolimus)	Calcineurin inhibition; steroid-sparing	Moderate	Off-label; widely used
Phototherapy (NB-UVB, PUVA, hand UVA)	Immunomodulation	Moderate	Established second-line
Delgocitinib (topical)	Pan-Janus kinase inhibition	High	Approved (EU 2024, US 2025)
Alitretinoin (oral)	Retinoid (9-cis-retinoic acid)	High	Approved for severe CHE; teratogenic
Dupilumab	Anti–IL-4Rα (blocks IL-4/IL-13)	Moderate	Off-label
Upadacitinib/abrocitinib (oral)	JAK1 inhibition	Low	Off-label

## 7. Management

Once occupational contact dermatitis is established, treatment proceeds along two tracks that must run in parallel: controlling the inflammation pharmacologically, and removing or reducing the exposure that provoked it. The second is easy to overlook in a clinical setting oriented toward prescribing, yet no topical or systemic agent will hold a disease in check if the hands return each day to the same unmodified insult. The therapeutic ladder set out below should therefore be read as sitting on top of the exposure-control and prevention measures of the previous section, not as a substitute for them. The principal therapeutic options, with their mechanisms and the strength of the evidence behind them, are summarised in Table 5.

### 7.1. Topical Therapy and Phototherapy

Topical corticosteroids remain the first-line pharmacological treatment for flares, and their role now carries a mechanistic footnote worth recalling: beyond suppressing inflammation, a two-week course measurably shifts the lesional microbiome back toward its non-lesional state, increasing diversity and reducing *Staphylococcus aureus* [56]. Topical calcineurin inhibitors, tacrolimus and pimecrolimus, provide a steroid-sparing alternative, useful where prolonged treatment risks the cutaneous atrophy that potent steroids can cause on repeated application. Atrophy is not, however, the adverse effect most likely to be missed. Contact sensitisation to the corticosteroid molecule itself was found in 2.7% of 6823 consecutively patch-tested patients in a Danish series, with tixocortol-21-pivalate, budesonide and hydrocortisone-17-butyrate serving as the baseline markers and about one third of sensitised patients co-reacting across corticosteroid classes [93]. Reactions to other molecules, mometasone furoate among them, are reported but are not reliably detected by the baseline markers alone, so that a negative baseline series does not exclude corticosteroid allergy. Because the anti-inflammatory action of the drug masks the very reaction it provokes, corticosteroid contact allergy should be suspected in any hand eczema that fails to improve, or paradoxically worsens, under an appropriately applied and adequately potent topical steroid; the appropriate response is an extended corticosteroid series with a delayed reading at day 7, together with testing for the common vehicle constituents, since sensitisation to preservatives, lanolin or fragrance in the base is at least as frequent as sensitisation to the active molecule. For chronic disease unresponsive to topical measures, phototherapy—narrowband UVB, oral or bath PUVA, and localised hand UVA—offers an established next step. Each of these modalities is positioned by severity within the German S2k guideline, which provides the stepwise escalation framework most European clinicians will follow [36].

The most consequential recent addition is delgocitinib, a first-in-class topical pan-Janus kinase inhibitor, now approved for moderate-to-severe chronic hand eczema in adults in the European Union (2024), the United States (2025), and several other markets [94,95]—the first topical agent licenced specifically for the indication. In the twin phase 3 DELTA 1 and DELTA 2 trials, treatment success on the Investigator’s Global Assessment at week 16 was achieved by roughly 20% of patients on delgocitinib versus 10% on vehicle in DELTA 1, and 29% versus 7% in DELTA 2 [23]. The open-label extension DELTA 3 showed responses maintained over up to 52 weeks of as-needed use, with a reassuring safety profile [24]. Results in adolescents (DELTA TEEN) have since been published in full: among 98 adolescents aged 12 to 17 years, 63.5% of those treated with delgocitinib achieved treatment success on the Investigator’s Global Assessment at week 16 against 29.2% on vehicle, and 71.6% reached a 90% improvement in the Hand Eczema Severity Index against 37.5%, with adverse events mostly mild to moderate and none serious [96]. Most consequentially, the head-to-head DELTA FORCE trial compared topical delgocitinib with oral alitretinoin—the only licenced systemic drug—in 513 adults with severe disease: delgocitinib produced a significantly greater reduction in HECSI at week 12 (least-squares mean change –67.6 versus –51.5; difference –16.1, 95% CI –23.3 to –8.9) and fewer adverse events (49% versus 76%) over 24 weeks [25].

### 7.2. Systemic Therapy

When topical therapy and phototherapy fail to control severe chronic hand eczema, systemic treatment becomes necessary, and here the options are more constrained than the burden of disease warrants. Alitretinoin, oral 9-cis-retinoic acid, remains the only systemic agent licenced specifically for severe chronic hand eczema refractory to potent topical corticosteroids, approved in Europe and Canada for courses of up to 24 weeks [26]. In the pivotal trial, up to 48% of patients achieved clear or almost-clear hands on the Physician’s Global Assessment, against 17% on placebo [26]. Its limitations are real, however: efficacy is variable between patients, the drug is potently teratogenic and therefore requires rigorous pregnancy prevention and monitoring in women of childbearing potential, and, as a recent editorial underlines, the lack of alternatives is particularly unfortunate when alitretinoin is ineffective or contraindicated [97]. This is the gap into which topical delgocitinib and the off-label biologic and small-molecule options have moved.

Dupilumab, the anti–IL-4-receptor-α monoclonal antibody, is used off-label and has the strongest mechanistic rationale of the biologics, given the Th2 suppression of antimicrobial peptides described earlier. A dedicated phase 2b, placebo-controlled, proof-of-concept trial demonstrated efficacy in patients with severe chronic hand eczema who had an inadequate response to, or intolerance of, alitretinoin [98], and a phase 3 randomised trial in atopic hand and foot dermatitis confirmed improvements in signs, symptoms, and quality of life [27]; observational and registry data point in the same direction. The oral Janus kinase inhibitors are the other emerging option: upadacitinib improved Hand Eczema Severity Index scores in patients with hand involvement in the two phase 3 atopic-dermatitis trials Measure Up 1 and 2, where percentage change in the index was a prespecified endpoint assessed at every visit and only the responder analysis was post hoc [99], and abrocitinib, an oral JAK1 inhibitor whose efficacy was established in a phase 2 randomised trial in atopic dermatitis [100], has more recently been reported to reduce severity in chronic hand eczema of diverse aetiology, including non-atopic disease, although those hand-eczema findings have so far been presented only in late-breaking conference form and await peer-reviewed publication—though both carry the class safety warnings that accompany systemic JAK inhibition and remain off-label for this indication. The reliance on off-label use across most of the systemic options underscores how recently the therapeutic landscape has begun to mature, and how much of current practice still runs ahead of formal licencing.

### 7.3. Protein Contact Dermatitis and Contact Urticaria

One occupationally important entity sits awkwardly within a discussion framed around irritant and allergic contact dermatitis, and it is worth treating separately because both its mechanism and its management diverge from everything described so far. Protein contact dermatitis is a chronic, recurrent eczema caused by high-molecular-weight proteins, in which acute exacerbations of itching and erythema appear within minutes of contact with the offending material, superimposed on a background of chronic hand dermatitis with scaling and fissures [101]. It is predominantly an occupational disease of food handlers—cooks, bakers, butchers, confectioners, fish and meat workers—and is similarly well recognised among veterinary surgeons, reflecting in each case repeated direct contact of the hands with fresh animal or plant proteins [102]. Inflammatory involvement of the nail folds, a paronychia-like change, is a useful clinical clue, as is the now-familiar pattern of improvement away from work and relapse on return.

The mechanistic point that matters for management is that protein contact dermatitis is driven, wholly or partly, by an immediate, IgE-mediated (type I) hypersensitivity rather than the delayed, T-cell-mediated mechanism of allergic contact dermatitis—sometimes with a type IV component superimposed [103]. This has a direct diagnostic consequence: standard delayed-reading patch tests are typically negative, and the diagnosis rests instead on immediate-type testing. Skin prick testing with the suspected protein-containing material is essential, and because cooking and processing denature the relevant allergens, fresh foods must be used—the prick-by-prick technique, in which the suspect food and then the patient’s forearm are pricked in immediate succession, is the practical method of choice, supplemented by specific IgE where available [104]. A meat sorter with negative standard patch tests but positive prick reactions to lamb and ox liver and to a blood mixture is a representative illustration of why the right test must be chosen for the right mechanism [105].

Management follows the same logic as the rest of occupational contact dermatitis but with the emphasis shifted decisively toward allergen identification and avoidance, because the prerequisite for successful treatment is identifying and excluding the eliciting allergen together with implementing skin protection [106]. Eliminating contact with the causative protein, through changes in food-handling practice and appropriate glove use, is therefore the cornerstone of management; the outcome data, however, temper any optimism about how easily this is achieved. In a questionnaire series of 178 patients with occupational food-related hand disease, those with protein contact dermatitis fared distinctly worse than the rest: 62.5% reported sick leave of more than three weeks against 30%, and 62% had to change job because of their skin against 43% [107]. Symptomatic flares are managed with topical anti-inflammatory therapy as for other forms of hand eczema, but no topical regimen substitutes for removing the protein contact. The relevance of all this to the post-pandemic setting is that the same immediate-type mechanism underlies contact urticaria to natural rubber latex, an exposure that rose sharply with intensified glove use among healthcare workers; the shift toward nitrile gloves, noted earlier as a barrier-sparing measure, doubles here as the means of avoiding a clinically distinct latex-driven reaction in sensitised individuals.

### 7.4. Return to Work

Pharmacological control is only half of management, and for occupational disease the more durable measure of success is whether the worker can remain in employment. The structured tertiary-prevention programmes discussed earlier are the clearest demonstration that this is achievable even in severe disease, with job retention of around 83% at three years in the German rehabilitation cohort [21]. Achieving it depends on a coordinated package that is largely independent of any particular drug. Workplace modification comes first—reducing the duration and intensity of wet work, substituting gentler agents, switching from latex to nitrile gloves, and introducing job rotation so that no single task dominates the working day. Where the eliciting agent is an identified allergen rather than a general irritant, workplace adaptation extends to removing or substituting that specific exposure, guided by patch-test results, and properly specified glove-and-emollient regimens, fitted to the tasks actually performed, complete the protective layer.

The occupational physician is central to this process, coordinating the assessment of fitness for work, the temporary modification of duties during flares, the implementation of skin-protection measures, and—in many jurisdictions—the formal recognition of the disease as occupational, which is often what unlocks access to structured rehabilitation and compensation. Timing matters: the longer severe disease persists before intervention, the lower the likelihood of remaining in the trade, which is why early referral is itself a determinant of job retention. Management, in this sense, closes the loop back to prevention. The same exposure-control measures that fail to show benefit as light-touch primary prevention in the healthy workforce become, when delivered intensively and in good time to a worker with established disease and a strong incentive to remain employed, part of a package that demonstrably keeps people in their jobs.

## 8. Gaps and Future Directions

**Figure 6:**
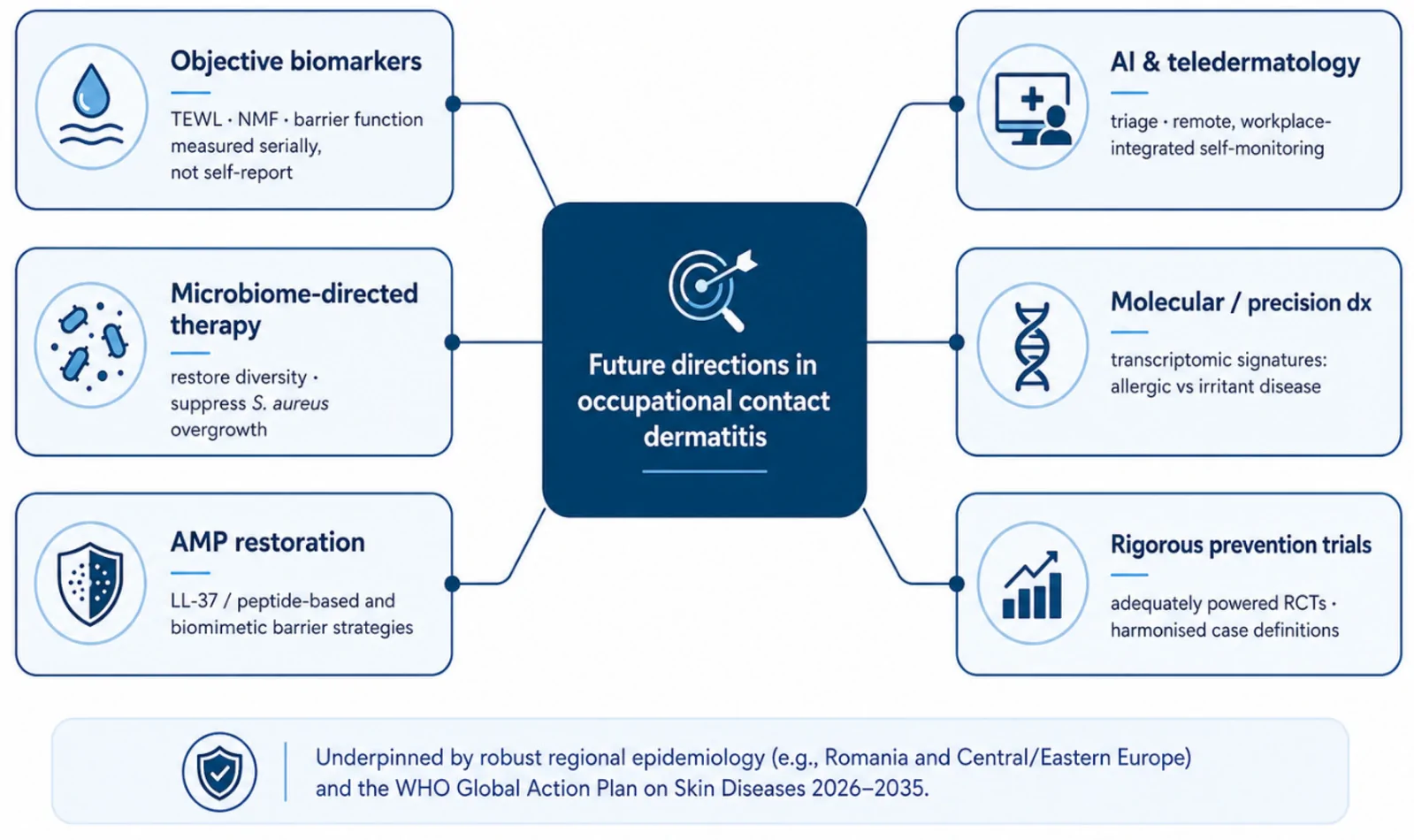
Priorities for future research and practice in occupational contact dermatitis. Six interlocking directions: objective barrier biomarkers (transepidermal water loss, natural moisturising factor) measured serially in place of self-report; microbiome-directed therapy to restore diversity and suppress *Staphylococcus aureus*; antimicrobial-peptide restoration and biomimetic barrier repair; AI-assisted triage and workplace-integrated teledermatology; molecular (transcriptomic) discrimination of allergic from irritant disease; and adequately powered prevention trials built on harmonised case definitions—all underpinned by robust regional epidemiology and the forthcoming WHO Global Action Plan on Skin Diseases.

If a single theme runs through this review, it is the mismatch between a rapidly deepening mechanistic understanding and a prevention evidence base that has not kept pace. That mismatch defines the research agenda, and several interlocking directions seem to us the most pressing. These priorities are summarised in Figure 6.

The first is methodological. The prevention literature is hampered less by a shortage of studies than by their heterogeneity—varying case definitions, inconsistent outcome measures, and high attrition—which is precisely why the 2018 Cochrane review could conclude only that barrier creams and moisturisers may help, on low-quality evidence [15]. The most glaring single gap is the complete absence of randomised trials of protective gloves, an intervention recommended almost universally yet never tested in this design [15]. What the field needs is not more small, idiosyncratic studies but adequately powered cluster trials built on harmonised occupational case definitions, validated severity scoring such as HECSI as a common endpoint, and objective barrier biomarkers—transepidermal water loss and natural moisturising factor chief among them—measured serially rather than relying on self-report. The null results of the Healthy Hands and PREVEX trials [16,17] should be read not as evidence that prevention is futile but as evidence that we have not yet designed the right interventions or measured them adequately, and the post hoc signal of possible harm in one subgroup [17] is itself an argument for the rigour that would let benefit and harm be distinguished with confidence.

The second direction follows directly from the pathophysiology. The barrier–dysbiosis–antimicrobial-peptide–inflammation loop set out earlier identifies several points of intervention that current prevention, focused as it is on occlusion and generic moisturisation, leaves untouched. Microbiome-directed strategies are the most concrete near-term prospect: approaches that suppress *Staphylococcus aureus* overgrowth or restore commensal diversity—including the use of commensal-derived antimicrobials such as the lantibiotics produced by coagulase-negative staphylococci—have shown promise in atopic dermatitis, where such antimicrobials are deficient [108], and are mechanistically rational in occupational disease, where the same staphylococcal overgrowth and the same correlation with severity have been documented directly in hand eczema [51,53]. The specific modalities, together with the parallel case for peptide-restoring strategies, are set out in Section 8.1 and are not repeated here. Formulation science is the more immediate translation: ceramide-dominant and biomimetic lamellar-lipid emollients [44], and acidic preparations that support the protease-restraining acid mantle [92], are mechanistically grounded in a way that simple occlusives are not, yet have never been tested head-to-head for occupational prevention in a high-certainty trial. This is, in our view, among the most answerable of the open questions, and one well suited to the kind of academic–industrial collaboration that translational dermatology increasingly depends on.

The third direction concerns how occupational skin disease is detected and monitored in the first place. The chronic underreporting documented earlier means many cases never enter surveillance at all, and the conventional model—periodic in-person dermatological assessment—scales poorly to large, dispersed, high-risk workforces. Teledermatology and artificial intelligence offer a plausible remedy: AI-based diagnostic-support tools have shown value as triage aids across large numbers of skin conditions, raising diagnostic agreement with specialists in primary-care settings [109], and teledermatology has matured into routine clinical service in several health systems. For occupational practice specifically, the attractive prospect is workplace-integrated self-monitoring and remote triage that could identify incident disease early, when intervention is most effective and job retention most achievable. Wearable sensors capable of continuously tracking barrier parameters could, in principle, bring this monitoring closer to real time. These tools remain early-stage for this application and require prospective validation in occupational cohorts rather than borrowed validation from general dermatology, but the direction of travel is clear. A related and nearer prospect is molecular diagnosis: the transcriptomic signatures that now distinguish allergic from irritant patch-test reactions [18] could, if validated in chronic clinical hand eczema rather than experimental settings, eventually convert the difficult irritant-versus-allergic judgement from an inference into a measurement.

The fourth direction is, frankly, an opportunity as much as a gap, and it bears directly on where this review originates. As set out in Section 3, robust epidemiological data on occupational skin disease are essentially absent for Romania and scarce across much of Central and Eastern Europe. This means prevention policy across a large part of Europe is being shaped without a clear picture of local burden, exposure patterns, or allergen prevalence. Well-designed regional prevalence and incidence studies—using validated instruments and, ideally, patch-test registry data—would not merely fill a descriptive void; they would provide the denominator against which any preventive intervention must be judged, and they sit squarely within the mandate of the World Health Organisation’s recently adopted recognition of skin diseases as a global public health priority and the forthcoming Global Action Plan on Skin Diseases it has initiated [4]. For research groups in the region, including those from which this review derives, that combination of a genuine evidence gap and a supportive global policy framework is an unusually clear invitation to contribute. The most useful first step would be a national registry of occupational skin disease—ideally linked to patch-test data, as the established networks in Germany and the Nordic countries have shown—converting scattered case series into a denominator-based picture of incidence, causative exposures, and outcomes. Beyond any single country, harmonised multicentre studies across Central and Eastern Europe, built on shared case definitions and a common occupational patch-test series, would let the region pool individually small datasets into evidence with real statistical and policy weight. Embedding such work within existing European contact-dermatitis networks would both accelerate it and ensure its findings are directly comparable with the Western European data that currently dominate the literature.

### 8.1. Emerging Therapeutic and Monitoring Strategies

The most direct translation of the pathophysiology is microbiome-targeted therapy. The observations that *S. aureus* overgrowth tracks with disease severity, and that commensal staphylococci secrete antimicrobials which selectively suppress it, have already moved bacteriotherapy from concept toward the clinic in atopic dermatitis, where first-in-human trials of an autologous commensal and of topically applied lactobacilli have now been reported [108,110,111]. Several modalities are plausible for occupational disease: topical probiotics and defined live biotherapeutic products applied to high-risk hands; autologous transplantation of a worker’s own commensal flora expanded ex vivo; and purified commensal-derived antimicrobials delivered in a barrier cream, suppressing *S. aureus* without the collateral damage of broad-spectrum antiseptics. The distinctive attraction in an occupational setting is prophylaxis rather than rescue—re-seeding and stabilising a healthy community on the hands of apprentices entering wet-work trades, before dysbiosis and disease take hold. What is missing is not mechanism but evidence: none of these approaches has yet been tested for occupational prevention, and the field needs dose-finding and controlled trials with both microbiological and clinical endpoints before any can be recommended.

Closely related is the prospect of antimicrobial-peptide replacement. Because LL-37 and the inducible β-defensins are expressed at reduced levels in the Th2-skewed state, in which the cytokine milieu prevents induction of these innate response genes [61,62], the skin is stripped of molecules that are simultaneously antimicrobial and barrier-reinforcing, LL-37 having been shown to upregulate tight-junction proteins and increase keratinocyte barrier function [59]; restoring peptide activity at the surface is therefore mechanistically compelling. Native peptides are unstable and expensive to manufacture, so the realistic routes are engineered LL-37 fragments with improved stability, small-molecule host-defence-peptide mimetics, and agents that induce endogenous peptide expression. For occupational skin specifically, the most original idea is to build the defence into the protective equipment itself: antimicrobial-peptide-coated or peptide-functionalised gloves and textiles that deliver activity precisely where exposure occurs—an approach that sits naturally alongside existing work on smart wound dressings and antimicrobial biomaterials. These strategies remain early-stage, with manufacturing, stability, and bacterial-resistance questions unresolved, but they trace a clear translational path from the barrier–dysbiosis–peptide loop to a deployable product.

If therapy is one frontier, measurement is the other. The prevention literature is hampered above all by its reliance on self-report and on single-timepoint clinical assessment, and wearable skin sensing offers a way out. Devices that quantify transepidermal water loss, hydration and electrical impedance are migrating from benchtop instruments toward miniaturised, wearable and textile-integrated formats capable of continuous monitoring, developed so far chiefly for atopic dermatitis severity [112,113]. Of particular relevance here, an epidermal sensing system has been reported for serine, one of the amino acids of the natural moisturising factor whose depletion was described in Section 4, which points toward direct, continuous readout of the barrier compartment that occupational exposures deplete [114]. In an occupational context this would convert barrier integrity from an occasional snapshot into a real-time signal—capturing the cumulative effect of a shift’s wet work or glove occlusion, flagging subclinical barrier impairment days before visible dermatitis, and supplying the objective, continuous outcome measure that prevention trials have so conspicuously lacked. The same data streams that would render a worker’s barrier visible in real time are also the raw material for prediction.

### 8.2. Risk Prediction and Precision Occupational Dermatology

Underlying all of this is a shift in how the disease itself is conceived. Hand eczema and occupational contact dermatitis are increasingly understood not as single entities but as clinically and molecularly heterogeneous conditions, in which irritant, allergic, and atopic contributions mix in proportions that differ from one patient to the next [115,116]. Transcriptomic profiling of chronic hand eczema has begun to make this concrete, identifying immune pathways shared across subtypes alongside molecular drivers specific to the irritant, allergic, and atopic forms [117]; the single-cell and spatial methods that reshaped atopic-dermatitis research are now being turned on the hand. If the disease is heterogeneous at the molecular level, a single preventive or therapeutic strategy applied to all comers is unlikely to be optimal for any—which is the essential argument for a precision approach.

Translating that insight into practice depends on biomarkers. A precision framework for occupational skin disease would draw on several classes at once: barrier biomarkers such as transepidermal water loss, stratum-corneum natural moisturising factor, and surface pH; the genetic-susceptibility marker of filaggrin loss-of-function status; microbiological markers such as *Staphylococcus aureus* load and microbiome diversity; molecular and inflammatory readouts, including the lesional transcriptomic signatures and circulating cytokine profiles now described for chronic hand eczema [117,118]; and emerging response-predictive biomarkers intended to match a given patient to a given drug [119]. The atopic-dermatitis field, further along this road, illustrates both the promise and the present limits of such markers—no single biomarker has yet been validated for routine clinical stratification [119,120,121]—yet recent work argues that molecular signatures may already begin to guide treatment selection in complex inflammatory skin disease [122]. The principal candidate biomarkers relevant to occupational contact dermatitis are summarised in Table 6.

Artificial intelligence is the natural complement to this proliferation of data. Multimodal foundation models trained across millions of dermatological images and associated data points are beginning to integrate the high-dimensional biomarker, transcriptomic, and exposure information that no clinician can synthesise unaided, supporting tasks that range from lesion classification to outcome prediction [123,124]; and for a field that must serve a diverse global workforce, the demonstration that such models can be engineered to perform more equitably across skin tones is especially pertinent [125].

Framed so far largely as a diagnostic aid, artificial intelligence already demonstrates clear value as a triage tool in primary care [109]. For occupational disease, the more transformative application is predictive rather than diagnostic. A model integrating exposure data (wet-work hours, glove time, irritant and allergen contact), host susceptibility (filaggrin genotype and atopic history), continuous barrier-sensor streams, and early patient-reported symptoms could estimate an individual worker’s risk of developing occupational contact dermatitis before clinical disease appears—identifying, within a cohort of apprentices, the minority for whom intensive primary prevention is genuinely worthwhile. This reframing matters because it addresses precisely why blanket, light-touch prevention has underperformed: interventions delivered indiscriminately to a largely low-risk population dilute their own measurable effect. Predictive stratification would let scarce preventive effort be concentrated where it actually changes outcomes. The accompanying questions—data governance, the ethics of pre-employment risk profiling, and the danger of excluding workers on the basis of genotype—are far from trivial and must be settled alongside the science, not after it. On filaggrin genotyping in particular, our position is deliberately restrictive, and we would not wish the preceding discussion to be read as advocacy for pre-employment screening. Loss-of-function carriage confers a modest odds ratio, has low positive predictive value at the level of the individual worker, and is neither necessary nor sufficient for disease: a substantial proportion of workers who develop occupational hand eczema are wild-type, and most carriers never develop it. Screening would therefore misclassify many workers while offering no protective intervention that is not already advisable for everyone entering a wet-work trade. It also raises unresolved questions of genetic discrimination in hiring and job allocation, worker autonomy and the right not to know, the confidentiality of genetic data held by an employer or an occupational health service, and compatibility with national occupational-health and data-protection law, which differs substantially between jurisdictions. We therefore do not recommend filaggrin genotyping as a screening tool in current occupational practice; its legitimate present uses are as a research variable and, at most, as one element of voluntary counselling offered to individuals who already have established disease.

Taken together, these strands point toward what might be called precision occupational dermatology: prevention and treatment matched to the individual worker’s barrier phenotype, filaggrin status, microbiome, and measured exposure, rather than applied uniformly. In practice this could mean accelerator-free gloves and a ceramide-dominant barrier regimen offered to the apprentice with a measurably vulnerable barrier phenotype from the first day of training; microbiome stabilisation for those already colonised by *S. aureus*; peptide-functionalised protective equipment for the highest-exposure tasks; and sensor-based monitoring to confirm that the chosen regimen is working—with the therapeutic ladder, now including topical delgocitinib, dupilumab for those with an atopic component [126], and the emerging systemic options, reserved for disease that is already established. None of this is guaranteed, and each component demands the rigorous, adequately powered evaluation this review has repeatedly argued is missing. But the trajectory is clear: the occupational dermatology of 2030 could move from generic creams applied after the fact toward biologically targeted, continuously monitored, and individually predicted prevention—and the regions that have historically lacked even basic epidemiological data, Romania and much of Central and Eastern Europe among them, are well placed to build that future-facing infrastructure from the ground up.

Realising this agenda will also require looking beyond the skin itself and situating occupational hand eczema within the wider practice of chronic-disease care. Accessible systemic markers of inflammation—such as the neutrophil-to-lymphocyte and platelet-to-lymphocyte ratios, which carry prognostic value across a range of inflammatory and surgical conditions [127]—offer a low-cost complement to the cutaneous and molecular biomarkers discussed above and merit formal evaluation in chronic occupational hand eczema. Where the clinical and non-invasive picture remains ambiguous, histopathological examination continues to provide a decisive confirmatory step, as it does throughout dermatological and surgical practice [128]. Better tests alone, however, will not close the persistent underreporting of occupational skin disease: patient-side barriers to seeking care, well documented across common conditions in which affected individuals delay or avoid presentation [129], must be addressed if surveillance is to reflect the true burden. And because that burden is as much psychosocial as physical, structured psychological support of the kind shown to improve quality of life in other chronic and disfiguring conditions [130] deserves an explicit place within comprehensive occupational care, alongside the barrier-, microbiome-, and immunity-directed strategies that form the core of this agenda.

## 9. Conclusions

Occupational contact dermatitis is the most common work-related skin disease, and it falls overwhelmingly on the hands. It is rarely dangerous and easily underestimated, yet it drives substantial cost, disrupts working lives, and pushes a meaningful share of those affected out of their trades. The COVID-19 pandemic made this visible by running, in effect, an involuntary experiment on the hands of the workforce—and the World Health Assembly’s 2025 recognition of skin diseases as a global public health priority, with a forthcoming Global Action Plan on Skin Diseases, finally gives the field the policy footing it has long lacked.

This review has argued that the field suffers from a specific and correctable asymmetry. The science of why occupational dermatitis develops has matured: the self-reinforcing loop linking barrier disruption, microbial dysbiosis, dysregulated antimicrobial peptides, and inflammation now gives chronicity a coherent mechanistic account, and molecular signatures are beginning to separate allergic from irritant disease at a level the bedside cannot reach. Treatment has matured too—delgocitinib is the first topical agent licenced for the indication and, in a head-to-head trial, outperformed the only systemic drug. Prevention has not kept pace. The strongest randomised trials of primary and secondary prevention have been essentially negative, the universally recommended protective glove has never been tested in a randomised design, and the most encouraging rehabilitation results rest on uncontrolled cohorts. The uncomfortable conclusion is that we understand this disease considerably better than we can prevent it.

We would put the diagnosis more pointedly: the prevention failure is less a failure of biology than of design and measurement. The interventions tested so far have largely been generic—apply a cream, attend a class—and were judged in good part by self-report, while the mechanisms uncovered over the past decade have scarcely been translated into what is actually trialled. The agenda that follows is therefore concrete. Prevention trials should be adequately powered, built on harmonised case definitions, and read out with objective barrier and microbial biomarkers rather than questionnaires. Formulations and microbiome- or peptide-directed strategies should be designed for the loop they aim to interrupt rather than around it. And the regions where the burden is real but the evidence absent—Romania and much of Central and Eastern Europe among them—should be mapped, because no intervention can be judged without a denominator. None of this is out of reach; what it requires is that the rigour now routine in describing this disease be turned, at last, toward preventing it. Occupational contact dermatitis should no longer be viewed solely as a disease of exposure, but as a dynamic disorder emerging from the interaction between barrier biology, microbial ecology, and innate immunity—and it is by acting on that interaction, not on exposure alone, that prevention will finally catch up with understanding.

Key Messages

- Occupational contact dermatitis is the commonest work-related skin disease, and its burden was intensified by COVID-19.
- A self-perpetuating barrier–dysbiosis–antimicrobial-peptide–inflammation cycle drives the disease toward chronicity.
- Primary-prevention evidence is weak: key randomised trials are null, and gloves remain untested in an RCT.
- Topical delgocitinib, superior to oral alitretinoin, is the first major therapeutic advance in decades.
- Future prevention should target barrier, microbiome, and immunity, guided by biomarkers and stronger regional data.

## Figures and Tables

**Table 1:** Principal studies informing this review, grouped by thematic domain. The list is illustrative of the higher-level evidence underpinning the synthesis rather than exhaustive. Studies were classified as principal, and therefore tabulated, when they met all three of the following criteria: (i) they represented the highest level of evidence available for their thematic domain (systematic review, meta-analysis, randomised controlled trial, or large registry or cohort study); (ii) they are cited in the corresponding section as the primary support for a substantive claim rather than as corroboration; and (iii) they report quantitative outcomes that can be summarised as a single key finding. Narrative reports, small case series, mechanistic in vitro studies and conference abstracts were excluded from the table even where they are cited in the text. CHE, chronic hand eczema; HECSI, Hand Eczema Severity Index; IGA-CHE, Investigator’s Global Assessment for chronic hand eczema; RCT, randomised controlled trial; GRADE, Grading of Recommendations Assessment, Development and Evaluation. Where certainty of evidence is stated in the key findings, it follows the four-level qualitative scale defined in the caption to Table 4.

Domain	Study (First Author, Year)	Design	Population/Sample	Key Finding
Epidemiology	Global Burden of Disease 2021 [5]	Global burden-of-disease modelling	Worldwide population	241 million prevalent dermatitis cases in 2021 (+38.8% vs. 1990); contact dermatitis the leading subtype (~253 million new cases per year).
Epidemiology	Larese Filon et al. [6]	Systematic review	Healthcare workers	Registry incidence 0.6–6.7 vs. cohort incidence 15.9–780.0 per 10,000 person-years—marked under-reporting, highest in apprentice nurses and dental staff.
Epidemiology	Lysdal et al. [19]	Register-based cohort	>5000 Danish hairdresser graduates	44.3% had left the trade after ~8 years; chronic hand eczema was more common among those who left.
Epidemiology	Polecka et al. [20]	Cross-sectional study	Polish working population	One-year hand-eczema prevalence 11.5% (women) and 6.7% (men); exacerbations linked to pandemic-era disinfectant use.
Prevention	Bauer et al., Cochrane review [15]	Systematic review of 9 RCTs (*n* = 2888)	Workers without hand dermatitis at baseline	Barrier creams and moisturisers may reduce incident irritant hand dermatitis; certainty low (GRADE).
Prevention	Skin-protection and secondary-prevention programmes [17,21,22]	RCT and multicentre cohort studies	Workers at risk of, or with, established occupational hand eczema	Structured education and tertiary rehabilitation improve disease course and occupational retention.
Treatment	Bissonnette et al., DELTA 1 & 2 [23]	Phase 3 vehicle-controlled RCTs	Adults, moderate-to-severe CHE	Topical delgocitinib: IGA-CHE treatment success at week 16 ~20% vs. 10% (DELTA 1) and 29% vs. 7% (DELTA 2).
Treatment	Gooderham et al., DELTA 3 [24]	Phase 3 open-label extension	Adults, CHE	Responses maintained over up to 52 weeks of as-needed use, with a reassuring safety profile.
Treatment	Giménez-Arnau et al., DELTA FORCE [25]	Head-to-head phase 3 RCT (*n* = 513)	Adults, severe CHE	Delgocitinib superior to oral alitretinoin: HECSI change at week 12 −67.6 vs. −51.5; fewer adverse events (49% vs. 76%).
Treatment	Ruzicka et al. [26]	Pivotal phase 3 RCT	Severe CHE refractory to topical corticosteroids	Oral alitretinoin produced clear/almost-clear hands in up to ~48% of patients vs. 17% on placebo.
Treatment	Simpson et al. [27]	Phase 3 RCT	Atopic hand and foot dermatitis	Dupilumab improved signs, symptoms, quality of life, and work productivity.

**Table 2:** Major occupational exposures associated with occupational contact dermatitis, by occupational group. Irritant exposures predominate in most groups; the allergens listed are those most frequently implicated on patch testing (or, for food proteins, on immediate-type testing) and are not exhaustive.

Occupation	Main Irritants	Main Allergens
Healthcare workers	Wet work, soaps and detergents, frequent handwashing, disinfectants, glove occlusion	Rubber accelerators (thiurams, carbamates), preservatives
Hairdressers	Shampoos, surfactants, bleaches, wet work	p-Phenylenediamine (PPD), persulphates, fragrances, nickel
Food handlers/catering	Wet work, friction, cleaning agents	Food proteins (immediate-type); spices, rubber additives
Construction/cement workers	Wet (alkaline) cement, abrasion	Chromate, cobalt, epoxy resins
Metalworkers	Cutting fluids, solvents, oils	Nickel, cobalt, biocides in metalworking fluids
Cleaners	Detergents, disinfectants, water, occlusive gloves	Isothiazolinones, fragrances, rubber accelerators

**Table 3:** The principal cutaneous antimicrobial peptides relevant to occupational contact dermatitis, with their main cellular sources, principal functions, and role in the barrier–dysbiosis–antimicrobial-peptide loop. hBD, human β-defensin; LL-37, cathelicidin-derived peptide; RNase 7, ribonuclease 7.

AMP	Source	Main Function	Role in Occupational Contact Dermatitis
LL-37 (cathelicidin)	Keratinocytes, neutrophils, eccrine glands	Broad-spectrum antimicrobial; chemotaxis; upregulates tight-junction proteins	Suppressed by Th2 cytokines, coupling barrier failure to *S. aureus* overgrowth; central node of the self-perpetuating loop
hBD-1	Keratinocytes (constitutive)	Constitutive baseline antimicrobial defence	Provides steady-state barrier immunity; relatively reduced in barrier-disrupted skin
hBD-2	Keratinocytes (inducible by IL-17/IL-22 and microbial signals)	Inducible antimicrobial, notably against Gram-negative bacteria and *Candida*	Induction blunted in the Th2-skewed milieu, impairing defence against colonisation
hBD-3	Keratinocytes (inducible)	Potent activity against *S. aureus*, including meticillin-resistant strains	Relative deficiency contributes to *S. aureus* susceptibility in eczematous skin
RNase 7	Keratinocytes, eccrine glands	Broad-spectrum constitutive antimicrobial ribonuclease	Key constituent of constitutive surface defence on frequently exposed skin
Psoriasin (S100A7)	Keratinocytes (high at exposed sites)	Antimicrobial, especially against *E. coli*; chemotactic	Surface defence on the hands; expression altered in inflamed, colonised skin
Dermcidin	Eccrine sweat glands	Constitutive, anionic sweat-derived antimicrobial	Contributes to sweat and acid-mantle defence; reduced output leaves fewer surface defences

**Table 4:** Summary of the evidence on the prevention of occupational hand dermatitis, with study design, population, effect, and certainty of evidence. Certainty of evidence is reported throughout this review on a single four-level qualitative scale. High denotes consistent evidence from adequately powered randomised controlled trials, or from systematic reviews and meta-analyses of such trials. Moderate denotes evidence from randomised trials with important limitations, or consistent evidence from large observational or registry studies. Low denotes evidence from single trials with important limitations, from small or single-centre studies, or from post hoc and subgroup analyses. Very low denotes uncontrolled cohorts, bodies of evidence too heterogeneous to be pooled or graded, and interventions supported by mechanistic rationale alone. Where a formal GRADE assessment was reported by the source, it is identified as such; all other ratings are the present authors’ assessment. The same scale is used in Table 1 and Table 5.

Intervention	Study/Design/Size	Population	Effect	Certainty
Barrier creams & moisturisers (primary prevention)	Cochrane systematic review 2018; 9 RCTs; *n* = 2888	Workers without irritant hand dermatitis at baseline	May reduce incident irritant hand dermatitis	Low (formal GRADE assessment)
Protective gloves	No randomised trials identified	—	Unknown—universally recommended yet never tested in an RCT	No evidence identified
Skin-care programme (Healthy Hands)	Cluster RCT; 19 wards; ~500 nurses	Hospital nurses	HECSI −6.2 vs. −4.2; not significant on primary outcome (significant only in mild-disease subgroup)	Low
Group educational programme (PREVEX)	Individually randomised RCT; *n* = 756	Newly notified occupational hand eczema	All three co-primary outcomes null; possible harm in healthcare-worker subgroup	Moderate
Inpatient multidisciplinary rehabilitation (ROQ)	Prospective cohort; *n* = 1410	Severe occupational skin disease; job at risk	82.7% job retention at 3 years; sustained reduction in severity	Very low
Secondary/tertiary prevention (Germany)	Systematic review; 19 studies; >5000 patients	Occupational skin disease	Consistently high job-retention rates	Very low
Hand-hygiene modality	Systematic review & meta-analysis	Workers/healthcare workers	Washing ≥8–10×/day: RR 1.51 (1.35–1.68); alcohol-based rub: not significant	Moderate
Ceramide-dominant/biomimetic emollients	Mechanistic + experimental/atopic data	Barrier-disrupted skin	Accelerate barrier repair; not tested for occupational prevention	Very low (rationale only)

**Table 6:** Candidate biomarkers for risk stratification and precision management of occupational contact dermatitis, grouped by category, with the typical measurement or sample and their potential clinical use. ACD, allergic contact dermatitis; ICD, irritant contact dermatitis; IR, infrared; NMF, natural moisturising factor; TEWL, transepidermal water loss. This table lists candidate biomarkers and does not grade certainty of evidence, since none of the markers has yet been validated for routine clinical use in occupational contact dermatitis; the potential clinical uses given are prospective rather than established.

Biomarker	Category	Measurement/Sample	Potential Clinical Use and Relevance
Filaggrin loss-of-function variants	Genetic susceptibility	Genotyping (blood or saliva)	Risk stratification—strongest known genetic risk factor for irritant susceptibility and chronicity; a research variable at present, and not recommended for pre-employment screening (Section 8.2)
Transepidermal water loss (TEWL)	Barrier function	Evaporimetry; emerging wearable sensors	Early detection—quantifies barrier integrity and rises before visible dermatitis; an objective, continuous outcome measure
Natural moisturising factor (NMF)	Barrier/corneocyte	Tape strips; Raman or IR spectroscopy	Barrier monitoring—reflects filaggrin breakdown products; reduced in barrier-impaired and filaggrin-deficient skin
Skin-surface pH	Barrier/acid mantle	Surface pH probe; emerging wearable sensors	Modifiable prevention target—elevated pH activates serine proteases and favours *S. aureus*
*S. aureus* load and microbiome diversity	Microbiological	Culture, qPCR, 16S or shotgun sequencing	Targeted prevention—overgrowth and reduced diversity track with disease severity; basis for microbiome-directed strategies
Antimicrobial peptides (LL-37, β-defensins)	Innate immunity	Tape strips or biopsy; immunoassay	Mechanistic and therapeutic target—suppressed in Th2-skewed skin, coupling barrier failure to colonisation
Lesional transcriptomic signature	Molecular/inflammatory	Skin biopsy; RNA sequencing	ICD vs. ACD discrimination—distinguishes irritant, allergic, and atopic subtypes; basis for endotype-driven therapy
Circulating cytokine and immune profile	Systemic inflammatory	Serum or plasma immunoassay	Non-invasive monitoring—associated with disease severity in chronic hand eczema
Blood eosinophils and total IgE	Atopic/type 2	Routine blood test	Endotype and response—mark the atopic endotype; may predict response to type-2-targeted therapy

## Data Availability

No new data were created or analysed in this study. Data sharing is not applicable to this article.

## Supplementary Materials

The following supporting information can be downloaded at: https://www.mdpi.com/article/10.3390/jcm15166353/s1. Supplementary Materials S1: Detailed database-specific search strategies, including the exact Boolean strings, field tags, subject headings and filters applied in each database.

## References

[1] Keegel T.G., Moyle M., Dharmage S., Frowen K., Nixon R. (2009). The Epidemiology of Occupational Contact Dermatitis (1990–2007): A Systematic Review. Int. J. Dermatol..

[2] Diepgen T.L., Coenraads P.J. (1999). The Epidemiology of Occupational Contact Dermatitis. Int. Arch. Occup. Environ. Health.

[3] Armstrong A., Hahn-Pedersen J., Bartlett C., Glanville J., Thyssen J.P. (2022). Economic Burden of Chronic Hand Eczema: A Review. Am. J. Clin. Dermatol..

[4] World Health Organization (2025). Skin Diseases as a Global Public Health Priority. Proceedings of the Seventy-Eighth World Health Assembly, Geneva, Switzerland, 27 May 2025.

[5] Fang J., Chen G., Wu M., Shi Y., Yuan C., Liu W. (2026). Trends and Projections of Dermatitis Burden (1990–2040): A 2021 Global Burden of Disease Analysis. Front. Med..

[6] Larese Filon F., Pesce M., Paulo M.S., Loney T., Modenese A., John S.M., Kezic S., Macan J. (2021). Incidence of Occupational Contact Dermatitis in Healthcare Workers: A Systematic Review. J. Eur. Acad. Dermatol. Venereol..

[7] Yüksel Y.T., Symanzik C., Christensen M.O., Olesen C.M., Thyssen J.P., Skudlik C., John S.M., Brans R., Agner T. (2024). Prevalence and Incidence of Hand Eczema in Healthcare Workers: A Systematic Review and Meta-Analysis. Contact Dermat..

[8] Quaade A.S., Simonsen A.B., Halling A.-S., Thyssen J.P., Johansen J.D. (2021). Prevalence, Incidence, and Severity of Hand Eczema in the General Population: A Systematic Review and Meta-Analysis. Contact Dermat..

[9] Loh E.D.W., Yew Y.W. (2022). Hand Hygiene and Hand Eczema: A Systematic Review and Meta-Analysis. Contact Dermat..

[10] Tatu A.L., Nadasdy T., Bujoreanu F.C. (2020). Familial Clustering of COVID-19 Skin Manifestations. Dermatol. Ther..

[11] Ma C., Sun J., Liu Z., Zhang C., Chen Y., Ma J., Ma Q. (2026). Skin Manifestations of Primary COVID-19 Infection with the Omicron Variant. PLoS ONE.

[12] Nguyen B.L., Cheng Y.J., Chua S.Y.I., Oh C.C. (2025). Trends in Dermatological Presentations at the Emergency Department of a Tertiary Hospital in Singapore: A 4-Year Analysis Pre- and during COVID-19. Int. J. Emerg. Med..

[13] Rudd E., Walsh S. (2021). Mask-Related Acne (“Maskne”) and Other Facial Dermatoses. BMJ.

[14] Yu J., Chen J.K., Mowad C.M., Reeder M., Hylwa S., Chisolm S., Dunnick C.A., Goldminz A.M., Jacon S.E., Wu P.A. (2021). Occupational Dermatitis to Facial Personal Protective Equipment in Health Care Workers: A Systematic Review. J. Am. Acad. Dermatol..

[15] Bauer A., Rönsch H., Elsner P., Dittmar D., Bennett C., Schuttelaar M.L.A., Lukács J., John S.M., Williams H.C. (2018). Interventions for Preventing Occupational Irritant Hand Dermatitis. Cochrane Database Syst. Rev..

[16] Soltanipoor M., Kezic S., Sluiter J.K., de Wit F., Bosma A.L., van Asperen R., Rustemeyer T. (2019). Effectiveness of a Skin Care Programme for the Prevention of Contact Dermatitis in Healthcare Workers (the Healthy Hands Project): A Single-Centre, Cluster Randomized Controlled Trial. Contact Dermat..

[17] Fisker M.H., Ebbehøj N.E., Vejlstrup S.G., Lindschou J., Gluud C., Winkel P., Bonde J.P., Agner T. (2018). Prevention of Hand Eczema: Effect of an Educational Program versus Treatment as Usual—Results of the Randomized Clinical PREVEX Trial. Scand. J. Work Environ. Health.

[18] Johansen J.D., Bonefeld C.M., Schwensen J.F.B., Thyssen J.P., Uter W. (2022). Novel Insights into Contact Dermatitis. J. Allergy Clin. Immunol..

[19] Lysdal S.H., Søsted H., Andersen K.E., Johansen J.D. (2011). Hand Eczema in Hairdressers: A Danish Register-Based Study of the Prevalence of Hand Eczema and Its Career Consequences. Contact Dermat..

[20] Polecka A., Awchimkow A., Owsianko N., Baran A., Hermanowicz J.M., Flisiak I. (2023). Hand Eczema in the Polish Female Population. J. Clin. Med..

[21] Brans R., Skudlik C., Weisshaar E., Scheidt R., Ofenloch R., Elsner P., Wulfhorst B., Schonfeld M., John S.M., Diepgen T.L. (2016). Multicentre Cohort Study ‘Rehabilitation of Occupational Skin Diseases—Optimization and Quality Assurance of Inpatient Management (ROQ)’: Results from a 3-Year Follow-Up. Contact Dermat..

[22] Ahlström M.G., Dietz J.B., Wilke A., Johansen J.D., John S.M., Brans R. (2022). Evaluation of the Secondary and Tertiary Prevention Strategies against Occupational Contact Dermatitis in Germany: A Systematic Review. Contact Dermat..

[23] Bissonnette R., Warren R.B., Pinter A., Agner T., Gooderham M., Schuttelaar M.L.A., Crépy M.N., Stingeni L., Serra-Baldrich E., Baranowski K. (2024). Efficacy and Safety of Delgocitinib Cream in Adults with Moderate to Severe Chronic Hand Eczema (DELTA 1 and DELTA 2): Results from Multicentre, Randomised, Controlled, Double-Blind, Phase 3 Trials. Lancet.

[24] Gooderham M., Molin S., Bissonnette R., Worm M., Crepy M.-N., Stingeni L., Warren R.B., Schliemann S., Schuttelaar M.-L., Ferrussi S. (2025). Long-Term Safety and Efficacy of Delgocitinib Cream for up to 52 Weeks in Adults with Chronic Hand Eczema: Results of the Phase 3 Open-Label Extension DELTA 3 Trial following the DELTA 1 and 2 trials. J. Am. Acad. Dermatol..

[25] Giménez-Arnau A.M., Pinter A., Sondermann W., Reguiai Z., Woolf R., Lynde C., Legat F.J., Costanzo A., Silvestre J., Mellerup N. (2025). Efficacy and Safety of Topical Delgocitinib Cream versus Oral Alitretinoin Capsules in Adults with Severe Chronic Hand Eczema (DELTA FORCE): A 24-Week, Randomised, Head-to-Head, Phase 3 Trial. Lancet.

[26] Ruzicka T., Lynde C.W., Jemec G.B.E., Diepgen T., Berth-Jones J., Coenraads P.J., Kaszuba A., Bissonnette R., Varjonen E., Hollo P. (2008). Efficacy and Safety of Oral Alitretinoin (9-cis Retinoic Acid) in Patients with Severe Chronic Hand Eczema Refractory to Topical Corticosteroids: Results of a Randomized, Double-Blind, Placebo-Controlled, Multicentre Trial. Br. J. Dermatol..

[27] Simpson E.L., Silverberg J.I., Worm M., Honari G., Masuda K., Syguła E., Schuttelaar M.L.A., Mortensen E., Laws E., Akinlade B. (2024). Dupilumab Treatment Improves Signs, Symptoms, Quality of Life, and Work Productivity in Patients with Atopic Hand and Foot Dermatitis: Results from a Phase 3, Randomized, Double-Blind, Placebo-Controlled Trial. J. Am. Acad. Dermatol..

[28] Pesqué D., Andrades E., Berenguer-Molins P., Perera-Bel J., Clarós M., Bódalo-Torruella M., González-Farré M., Gallardo F., Pujol R.M., Giménez-Arnau A.M. (2025). Transcriptomic Analysis of Allergic Patch Test Reactions in Non-Atopic Patients: A Comparative Study Across Multiple Allergens. Allergy.

[29] Alinaghi F., Havmose M., Thyssen J.P., Zachariae C., Johansen J.D. (2023). Contact Allergy to Metals in Metalworkers: A Systematic Review and Meta-Analysis. Contact Dermat..

[30] Sedeh F.B., Michaelsdóttir T.E., Jemec G.B.E., Mortensen O.S., Ibler K.S. (2023). Prevalence, Risk Factors, and Prevention of Occupational Contact Dermatitis among Professional Cleaners: A Systematic Review. Int. Arch. Occup. Environ. Health.

[31] Kridin K., Bergman R., Khamaisi M., Zelber-Sagi S., Weltfriend S. (2016). Cement-Induced Chromate Occupational Allergic Contact Dermatitis. Dermatitis.

[32] Jamil W., Svensson Å., Josefson A., Lindberg M., von Kobyletzki L. (2022). Incidence Rate of Hand Eczema in Different Occupations: A Systematic Review and Meta-Analysis. Acta Derm.-Venereol..

[33] Chiriac A.E., Azoicai D., Chiriac A., Naznean A., Larese Filon F., Georgescu S.R., Foia L., Podoleanu C., Stolnicu S. (2016). Romanian Questionnaire to Assess the Prevalence of Occupational Hand Eczema among Healthcare Providers. J. Interdiscip. Med..

[34] Moldovan H.R., Voidazan S.T., John S.M., Weinert P., Moldovan G., Vlasiu M.A., Szasz Z.A., Tiplica G.S., Szasz S., Marin A.C. (2017). The Eastern European Experience on Occupational Skin Diseases. Make Underreporting an Issue?. J. Eur. Acad. Dermatol. Venereol..

[35] Isufi D., Kursawe Larsen C., Jensen M.B., Karimian K., Zachariae C., Schwensen J.F.B., Alinaghi F., Johansen J.D. (2025). Prevalence of Contact Allergy to Rubber Accelerators from the European Baseline Series in Dermatitis Patients: A Systematic Review and Meta-Analysis. Contact Dermat..

[36] Bauer A., Brans R., Brehler R., Büttner M., Elsner P., Fartasch M., Herzog C., John S.-M., Kollner A., Maul J.-T. (2023). S2k Guideline: Diagnosis, Prevention, and Therapy of Hand Eczema. J. Dtsch. Dermatol. Ges..

[37] Kezic S., O’Regan G.M., Yau N., Sandilands A., Chen H., Campbell L.E., Kroboth K., Watson R., Rowland M., McLean W.H.I. (2011). Levels of Filaggrin Degradation Products Are Influenced by Both Filaggrin Genotype and Atopic Dermatitis Severity. Allergy.

[38] Palmer C.N.A., Irvine A.D., Terron-Kwiatkowski A., Zhao Y.W., Liao H.H., Lee S.P., Goudie D.R., Sandilands A., Campbell L.E., Smith F.J.D. (2006). Common Loss-of-Function Variants of the Epidermal Barrier Protein Filaggrin Are a Major Predisposing Factor for Atopic Dermatitis. Nat. Genet..

[39] de Jongh C.M., Khrenova L., Verberk M.M., Calkoen F., Van Dijk F.J.H., Voss H., John S.M., Kezic S. (2008). Loss-of-Function Polymorphisms in the Filaggrin Gene Are Associated with an Increased Susceptibility to Chronic Irritant Contact Dermatitis: A Case–Control Study. Br. J. Dermatol..

[40] Visser M.J., Landeck L., Campbell L.E., McLean W.H.I., Weidinger S., Calkoen F., John S.M., Kezic S. (2013). Impact of Atopic Dermatitis and Loss-of-Function Mutations in the Filaggrin Gene on the Development of Occupational Irritant Contact Dermatitis. Br. J. Dermatol..

[41] Timmerman J.G., Heederik D., Spee T., van Rooy F.G., Krop E.J.M., Koppelman G.H., Rustemeyer T., Smit L.A.M. (2016). Contact Dermatitis in the Construction Industry: The Role of Filaggrin Loss-of-Function Mutations. Br. J. Dermatol..

[42] Bandier J., Ross-Hansen K., Carlsen B.C., Menné T., Linneberg A., Stender S., Szecsi P.B., Meldgaard M., Thyssen J.P., Johansen J.D. (2013). Carriers of Filaggrin Gene (FLG) Mutations Avoid Professional Exposure to Irritants in Adulthood. Contact Dermat..

[43] Janssens M., van Smeden J., Gooris G.S., Bras W., Portale G., Caspers P.J., Vreeken R.J., Hankemeier T., Kezic S., Wolterbeek R. (2012). Increase in Short-Chain Ceramides Correlates with an Altered Lipid Organization and Decreased Barrier Function in Atopic Eczema Patients. J. Lipid Res..

[44] Andrew P.V., Williams S.F., Brown K., Chittock J., Pinnock A., Poyner A., Cork M.J., Danby S.G. (2025). Topical Supplementation with Physiological Lipids Rebalances the Stratum Corneum Ceramide Profile and Strengthens Skin Barrier Function in Adults Predisposed to Atopic Dermatitis. Br. J. Dermatol..

[45] Deraison C., Bonnart C., Lopez F., Besson C., Robinson R., Jayakumar A., Wagberg F., Brattsand M., Hachem J.P., Leonardsson G. (2007). LEKTI Fragments Specifically Inhibit KLK5, KLK7, and KLK14 and Control Desquamation through a pH-Dependent Interaction. Mol. Biol. Cell.

[46] Chavanas S., Bodemer C., Rochat A., Hamel-Teillac D., Ali M., Irvine A.D., Bonafe J.-L., Wilkinson J., Taieb A., Barrandon Y. (2000). Mutations in SPINK5, Encoding a Serine Protease Inhibitor, Cause Netherton Syndrome. Nat. Genet..

[47] De Benedetto A., Rafaels N.M., McGirt L.Y., Ivanon A.I., Georas S.N., Cheadle C., Berger A.E., Zhang K., Vidyasagar S., Yoshida T. (2011). Tight Junction Defects in Patients with Atopic Dermatitis. J. Allergy Clin. Immunol..

[48] Koppes S.A., Ljubojević Hadžavdić S., Jakasa I., Franceschi N., Riethmüller C., Jurakić Tončić R., Marinović B., Raj N., Rawlings A.V., Voegeli R. (2017). Effect of Allergens and Irritants on Levels of Natural Moisturizing Factor and Corneocyte Morphology. Contact Dermat..

[49] Schmid K., Broding H.C., Uter W., Drexler H. (2005). Transepidermal Water Loss and Incidence of Hand Dermatitis in a Prospectively Followed Cohort of Apprentice Nurses. Contact Dermat..

[50] Byrd A.L., Belkaid Y., Segre J.A. (2018). The Human Skin Microbiome. Nat. Rev. Microbiol..

[51] Nørreslet L.B., Edslev S.M., Andersen P.S., Plum F., Holt J., Kjerulf A., Ebbehøj N.E., Clausen M.L., Flachs E.M., Agner T. (2020). Colonization with *Staphylococcus aureus* in Patients with Hand Eczema: Prevalence and Association with Severity, Atopic Dermatitis, Subtype and Nasal Colonization. Contact Dermat..

[52] Nørreslet L.B., Edslev S.M., Clausen M.L., Flachs E.M., Ebbehøj N.E., Andersen P.S., Agner T. (2022). Hand Eczema and Temporal Variation of *Staphylococcus aureus* Clonal Complexes: A Prospective Observational Study. J. Am. Acad. Dermatol..

[53] Nørreslet L.B., Lilje B., Ingham A.C., Edslev S.M., Clausen M.L., Plum F., Andersen P.S., Agner T. (2022). Skin Microbiome in Patients with Hand Eczema and Healthy Controls: A Three-Week Prospective Study. Acta Derm. Venereol..

[54] Vindenes H.K., Drengenes C., Amin H., Irgens-Hansen K., Svanes C., Bertelsen R.J. (2024). Longitudinal Analysis of the Skin Microbiome in Association with Hand Eczema, Hand Hygiene Practices and Moisturizer Use. J. Eur. Acad. Dermatol. Venereol..

[55] Miajlovic H., Fallon P.G., Irvine A.D., Foster T.J. (2010). Effect of Filaggrin Breakdown Products on Growth of and Protein Expression by *Staphylococcus aureus*. J. Allergy Clin. Immunol..

[56] Nørreslet L.B., Ingham A.C., Agner T., Olesen C.M., Bregnhøj A., Sommerlund M., Andersen P.S., Stegger M., Mørtz C.G., Edslev S.M. (2025). Hand Eczema and Changes in the Skin Microbiome after 2 Weeks of Topical Corticosteroid Treatment. J. Eur. Acad. Dermatol. Venereol..

[57] Watanabe H., Gaide O., Pétrilli V., Martinon F., Contassot E., Roques S., Kummer J.A., Tschopp J., French L.E. (2007). Activation of the IL-1β-Processing Inflammasome Is Involved in Contact Hypersensitivity. J. Investig. Dermatol..

[58] Weber F.C., Esser P.R., Müller T., Ganesan J., Pellegatti P., Simon M.M., Zeiser R., Idzko M., Jakob T., Martin S.F. (2010). Lack of the Purinergic Receptor P2X7 Results in Resistance to Contact Hypersensitivity. J. Exp. Med..

[59] Akiyama T., Niyonsaba F., Kiatsurayanon C., Nguyen T.T., Ushio H., Fujimura T., Ueno T., Okumura K., Ogawa H., Ikeda S. (2014). The Human Cathelicidin LL-37 Host Defense Peptide Upregulates Tight Junction-Related Proteins and Increases Human Epidermal Keratinocyte Barrier Function. J. Innate Immun..

[60] Harder J., Schröder J.M., Gläser R. (2013). The Skin Surface as Antimicrobial Barrier: Present Concepts and Future Outlooks. Exp. Dermatol..

[61] Ong P.Y., Ohtake T., Brandt C., Strickland I., Boguniewicz M., Ganz T., Gallo R.L., Leung D.Y.M. (2002). Endogenous Antimicrobial Peptides and Skin Infections in Atopic Dermatitis. N. Engl. J. Med..

[62] Nomura I., Goleva E., Howell M.D., Hamid Q.A., Ong P.Y., Hall C.F., Darst M.A., Gao B., Boguniewicz M., Travers J.B. (2003). Cytokine Milieu of Atopic Dermatitis, as Compared to Psoriasis, Skin Prevents Induction of Innate Immune Response Genes. J. Immunol..

[63] Williams M.R., Costa S.K., Zaramela L.S., Khalil S., Todd D.A., Winter H.L., Sanford J.A., O’Neill A.M., Liggins M.C., Nakatsuji T. (2019). Quorum Sensing between Bacterial Species on the Skin Protects against Epidermal Injury in Atopic Dermatitis. Sci. Transl. Med..

[64] Yamasaki K., Di Nardo A., Bardan A., Murakami M., Ohtake T., Coda A., Dorschner R.A., Bonnart C., Descargues P., Hovnanian A. (2007). Increased Serine Protease Activity and Cathelicidin Promotes Skin Inflammation in Rosacea. Nat. Med..

[65] Rippke F., Schreiner V., Doering T., Maibach H.I. (2004). Stratum Corneum pH in Atopic Dermatitis: Impact on Skin Barrier Function and Colonization with *Staphylococcus aureus*. Am. J. Clin. Dermatol..

[66] Williams M.R., Nakatsuji T., Sanford J.A., Vrbanac A.F., Gallo R.L. (2017). *Staphylococcus aureus* Induces Increased Serine Protease Activity in Keratinocytes. J. Investig. Dermatol..

[67] Gryllos I., Tran-Winkler H.J., Cheng M.-F., Chung H., Bolcome R., Lu W., Lehrer R.I., Wessels M.R. (2008). Induction of Group A Streptococcus Virulence by a Human Antimicrobial Peptide. Proc. Natl. Acad. Sci. USA.

[68] Velarde J.J., Ashbaugh M., Wessels M.R. (2014). The Human Antimicrobial Peptide LL-37 Binds Directly to CsrS, a Sensor Histidine Kinase of Group A Streptococcus, to Activate Expression of Virulence Factors. J. Biol. Chem..

[69] Demirci M., Yigin A., Demir C. (2022). Efficacy of Antimicrobial Peptide LL-37 against Biofilm-Forming *Staphylococcus aureus* Strains Obtained from Chronic Wound Infections. Microb. Pathog..

[70] Sonesson A., Przybyszewska K., Eriksson S., Mörgelin M., Kjellström S., Davies J., Potempa J., Schmidtchen A. (2017). Identification of Bacterial Biofilm and the *Staphylococcus aureus*-Derived Protease, Staphopain, on the Skin Surface of Patients with Atopic Dermatitis. Sci. Rep..

[71] Otto M. (2023). Critical Assessment of the Prospects of Quorum-Quenching Therapy for *Staphylococcus aureus* Infection. Int. J. Mol. Sci..

[72] Yamaguchi H.L., Yamaguchi Y., Peeva E. (2023). Role of Innate Immunity in Allergic Contact Dermatitis: An Update. Int. J. Mol. Sci..

[73] Kaplan D.H., Igyártó B.Z., Gaspari A.A. (2012). Early Immune Events in the Induction of Allergic Contact Dermatitis. Nat. Rev. Immunol..

[74] Vocanson M., Hennino A., Rozières A., Poyet G., Nicolas J.F. (2009). Effector and Regulatory Mechanisms in Allergic Contact Dermatitis. Allergy.

[75] Kehren J., Desvignes C., Krasteva M., Ducluzeau M.T., Assossou O., Horand F., Hahne M., Kägi D., Kaiserlian D., Nicolas J.F. (1999). Cytotoxicity Is Mandatory for CD8+ T Cell-Mediated Contact Hypersensitivity. J. Exp. Med..

[76] Ring S., Oliver S.J., Cronstein B.N., Enk A.H., Mahnke K. (2009). CD4+CD25+ Regulatory T Cells Suppress Contact Hypersensitivity Reactions through a CD39, Adenosine-Dependent Mechanism. J. Allergy Clin. Immunol..

[77] Frisoli M.L., Ko W.C.C., Martinez N., Afshari K., Wang Y., Garber M., Harris J.E. (2025). Single-Cell RNA Sequencing Reveals Molecular Signatures That Distinguish Allergic from Irritant Contact Dermatitis. J. Investig. Dermatol..

[78] Fortino V., Wisgrill L., Werner P., Suomela S., Linder N., Jalonen E., Suomalainen A., Marwah V., Kero M., Pesonen M. (2020). Machine-Learning-Driven Biomarker Discovery for the Discrimination between Allergic and Irritant Contact Dermatitis. Proc. Natl. Acad. Sci. USA.

[79] Lefevre M.-A., Nosbaum A., Mosnier A., Lenief V., Salque S., Pichot M., Maheux L., Bertolotti L., Hacard F., Graveriau C. (2024). Gene Profiling in Active Dermatitis Lesions Strengthens the Diagnosis of Allergic Contact Dermatitis. J. Am. Acad. Dermatol..

[80] Schmidt M., Raghavan B., Müller V., Vogl T., Fejer G., Tchaptchet S., Keck S., Kalis C., Nielsen P.J., Galanos C. (2010). Crucial Role for Human Toll-like Receptor 4 in the Development of Contact Allergy to Nickel. Nat. Immunol..

[81] Adam C., Wohlfarth J., Haußmann M., Sennefelder H., Rodin A., Maler M., Martin S.F., Goebeler M., Schmidt M. (2017). Allergy-Inducing Chromium Compounds Trigger Potent Innate Immune Stimulation via ROS-Dependent Inflammasome Activation. J. Investig. Dermatol..

[82] Aeby P., Sieber T., Beck H., Gerberick G.F., Goebel C. (2009). Skin Sensitization to p-Phenylenediamine: The Diverging Roles of Oxidation and N-Acetylation for Dendritic Cell Activation and the Immune Response. J. Investig. Dermatol..

[83] Alvarez-Sánchez R., Basketter D., Pease C., Lepoittevin J.-P. (2003). Studies of Chemical Selectivity of Hapten, Reactivity, and Skin Sensitization Potency. 3. Synthesis and Studies on the Reactivity toward Model Nucleophiles of the 13C-Labeled Skin Sensitizers 5-Chloro-2-methylisothiazol-3-one and 2-Methylisothiazol-3-one. Chem. Res. Toxicol..

[84] Aleksic M., Meng X. (2024). Protein Haptenation and Its Role in Allergy. Chem. Res. Toxicol..

[85] Held E., Skoet R., Johansen J.D., Agner T. (2005). The Hand Eczema Severity Index (HECSI): A Scoring System for Clinical Assessment of Hand Eczema. A Study of Inter- and Intraobserver Reliability. Br. J. Dermatol..

[86] Astner S., González S., Gonzalez E. (2006). Noninvasive Evaluation of Allergic and Irritant Contact Dermatitis by In Vivo Reflectance Confocal Microscopy. Dermatitis.

[87] Boone M.A.L.M., Jemec G.B.E., Del Marmol V. (2015). Differentiating Allergic and Irritant Contact Dermatitis by High-Definition Optical Coherence Tomography: A Pilot Study. Arch. Dermatol. Res..

[88] Ruini C., Hartmann D., Rahimi F., Fiocco Z., French L.E., Oppel E., Sattler E. (2021). Optical Coherence Tomography for Patch Test Grading: A Prospective Study on Its Use for Noninvasive Diagnosis of Allergic Contact Dermatitis. Contact Dermat..

[89] Stocks S.J., McNamee R., Turner S., Carder M., Agius R.M. (2012). Has European Union Legislation to Reduce Exposure to Chromate in Cement Been Effective in Reducing the Incidence of Allergic Contact Dermatitis Attributed to Chromate in the UK?. Occup. Environ. Med..

[90] Bensefa-Colas L., Stocks S.J., McNamee R., Faye S., Pontin F., Agius R.M., Lasfargues G., Telle-Lamberton M., Momas I. (2017). Effectiveness of the European Chromium(VI) Directive for Cement Implementation on Occupational Allergic Contact Dermatitis Occurrence: Assessment in France and the U.K. Br. J. Dermatol..

[91] Loi A.S.T., Aribou Z.M., Fong Y.T. (2022). Improving Recovery of Irritant Hand Dermatitis in Healthcare Workers with Workplace Interventions during the COVID-19 Pandemic. Front. Public Health.

[92] Andrew P.V., Pinnock A., Poyner A., Brown K., Chittock J., Kay L.J., Cork M.J., Danby S.G. (2024). Maintenance of an Acidic Skin Surface with a Novel Zinc Lactobionate Emollient Preparation Improves Skin Barrier Function in Patients with Atopic Dermatitis. Dermatol. Ther..

[93] Svendsen S.V., Bach R.O., Mortz C.G. (2022). Prevalence of Contact Allergy to Corticosteroids in a Danish Patient Population. Contact Dermat..

[94] European Medicines Agency (2024). Anzupgo: EPAR—Product Information.

[95] U.S. Food and Drug Administration (2025). ANZUPGO (Delgocitinib) Cream: Prescribing Information.

[96] Molin S., Baselga E., Navarro-Triviño F.J., Reguiai Z., De Schepper S., Halpern J., Marcoux D., Su J.C., Brzewski P., Antonsen S.D. (2026). Delgocitinib Cream for Adolescents with Moderate to Severe Chronic Hand Eczema (DELTA TEEN): A Multicentre, Double-Blind, Phase 3 Randomised Controlled Trial. Lancet Child Adolesc. Health.

[97] Brans R. (2023). Promising Results for Treatment of Severe Chronic Hand Eczema with Dupilumab. Br. J. Dermatol..

[98] Voorberg A.N., Kamphuis E., Christoffers W.A., Schuttelaar M.L.A. (2023). Efficacy and Safety of Dupilumab in Patients with Severe Chronic Hand Eczema with Inadequate Response or Intolerance to Alitretinoin: A Randomized, Double-Blind, Placebo-Controlled Phase IIb Proof-of-Concept Study. Br. J. Dermatol..

[99] Simpson E.L., Rahawi K., Hu X., Chu A.D., Nduaka C., Jazayeri S., Lio P., Lynde C., Schuttelaar M.L.A. (2023). Effect of Upadacitinib on Atopic Hand Eczema in Patients with Moderate-to-Severe Atopic Dermatitis: Results from Two Randomized Phase 3 Trials. J. Eur. Acad. Dermatol. Venereol..

[100] Gooderham M.J., Forman S.B., Bissonnette R., Beebe J.S., Zhang W., Banfield C., Zhu L., Papacharalambous J., Vincent M.S., Peeva E. (2019). Efficacy and Safety of Oral Janus Kinase 1 Inhibitor Abrocitinib for Patients with Atopic Dermatitis: A Phase 2 Randomized Clinical Trial. JAMA Dermatol..

[101] Rocha J., Pereira T., Basto A.S., Brito C. (2010). Occupational Protein Contact Dermatitis: Two Case Reports. Case Rep. Med..

[102] Mahler V., Johansen J.D., Mahler V., Lepoittevin J.-P., Frosch P.J. (2020). Occupational Contact Dermatitis: Chefs and Food Handlers. Contact Dermatitis.

[103] Barbaud A., Poreaux C., Penven E., Waton J. (2015). Occupational Protein Contact Dermatitis. Eur. J. Dermatol..

[104] Barbaud A. (2020). Mechanism and Diagnosis of Protein Contact Dermatitis. Curr. Opin. Allergy Clin. Immunol..

[105] Kumar A., Freeman S. (1999). Protein Contact Dermatitis in Food Workers: Case Report of a Meat Sorter and Summary of Seven Other Cases. Australas. J. Dermatol..

[106] Levin C., Warshaw E. (2008). Protein Contact Dermatitis: Allergens, Pathogenesis, and Management. Dermatitis.

[107] Vester L., Thyssen J.P., Menné T., Johansen J.D. (2012). Consequences of Occupational Food-Related Hand Dermatoses with a Focus on Protein Contact Dermatitis. Contact Dermat..

[108] Nakatsuji T., Chen T.H., Narala S., Chun K.A., Two A., Yun T., Shafiq F., Kotol P.F., Bouslimani A., Melnik A.V. (2017). Antimicrobials from Human Skin Commensal Bacteria Protect against *Staphylococcus aureus* and Are Deficient in Atopic Dermatitis. Sci. Transl. Med..

[109] Jain A., Way D., Gupta V., Gao Y., de Oliveira Marinho G., Hartford J., Sayres R., Kanada K., Eng C., Nagpal K. (2021). Development and Assessment of an Artificial Intelligence-Based Tool for Skin Condition Diagnosis by Primary Care Physicians and Nurse Practitioners in Teledermatology Practices. JAMA Netw. Open.

[110] Nakatsuji T., Hata T.R., Tong Y., Cheng J.Y., Shafiq F., Butcher A.M., Salem S.S., Brinton S.L., Rudman Spergel A.K., Johnson K. (2021). Development of a Human Skin Commensal Microbe for Bacteriotherapy of Atopic Dermatitis and Use in a Phase 1 Randomized Clinical Trial. Nat. Med..

[111] Lebeer S., Oerlemans E., Claes I., Henkes T., Delanghe L., Wuyts S., Spacova I., van den Broek M.F.L., Tuyaerts I., Wittouck S. (2022). Selective Targeting of Skin Pathobionts and Inflammation with Topically Applied Lactobacilli. Cell Rep. Med..

[112] Khan Y., Todorov A., Torah R., Beeby S., Ardern-Jones M.R. (2024). Skin Sensing and Wearable Technology as Tools to Measure Atopic Dermatitis Severity. Ski. Health Dis..

[113] Matsui N., Ohnishi A., Uyama A., Sugino T., Terada T., Tsukamoto M. (2024). Wearable System for Continuous Estimation of Transepidermal Water Loss. Electronics.

[114] Yuan Y., Zhong B., Qin X., Xu H., Li Z., Li L., Wang X., Zhang W., Lou Z., Fan Y. (2025). An Epidermal Serine Sensing System for Skin Healthcare. Nat. Commun..

[115] Weidinger S., Novak N. (2024). Hand Eczema. Lancet.

[116] Karagounis T.K., Cohen D.E. (2023). Occupational Hand Dermatitis. Curr. Allergy Asthma Rep..

[117] Quaade A.S., Litman T., Wang X., Becker C., McCauley B.D., Sølberg J.B.K., Thyssen J.P., Johansen J.D. (2025). Transcriptomic Profiling of Chronic Hand Eczema Skin Reveals Shared Immune Pathways and Molecular Drivers across Subtypes. J. Allergy Clin. Immunol..

[118] Tauber M., Vocanson M. (2024). Biomarkers for Chronic Hand Eczema: Could Etiological and Clinical Subtypes Truly Be the Starting Point? An Open Question. J. Eur. Acad. Dermatol. Venereol..

[119] Renert-Yuval Y., Thyssen J.P., Bissonnette R., Bieber T., Kabashima K., Hijnen D., Guttman-Yassky E. (2021). Biomarkers in Atopic Dermatitis—A Review on Behalf of the International Eczema Council. J. Allergy Clin. Immunol..

[120] Chiricozzi A., Levi A., Palladino C., Girolomoni G. (2025). Enabling Precision Medicine with Biomarkers of Response to Treatment in Atopic Dermatitis: Where Are We Now? A Narrative Review. Front. Med..

[121] Bieber T. (2025). The Paradigm Shift in Drug Development for Atopic Dermatitis. Ann. Allergy Asthma Immunol..

[122] Beaulieu V., Mosnier A., Rimet H., Garreau A.-C., Jaulent-Darmaisin C., Jaulent L., Fathallah R., Berard F., Hacard F., Nicolas J.-F. (2026). Skin’s Molecular Signature May Help Guide Treatment of Complex Inflammatory Skin Diseases. J. Eur. Acad. Dermatol. Venereol..

[123] Tang A.S., Wei M.L., Haemel A., La C., Sirota M., Lee E.Y. (2026). Artificial Intelligence-Enabled Precision Medicine for Inflammatory Skin Diseases. J. Investig. Dermatol..

[124] Yan S., Yu Z., Primiero C., Vico-Alonso C., Wang Z., Yang L., Tschandl P., Hu M., Ju G., Tan G. (2025). A Multimodal Vision Foundation Model for Clinical Dermatology. Nat. Med..

[125] Groh M., Badri O., Daneshjou R., Koochek A., Harris C., Soenksen L.R., Doraiswamy P.M., Picard R. (2024). Deep Learning-Aided Decision Support for Diagnosis of Skin Disease across Skin Tones. Nat. Med..

[126] Riva H.R., Yoon T., Hendricks A.J., Malick H., Chisholm C.D., Housewright C.D., Singh V., Guevara A. (2026). Dupilumab for Chronic Hand Eczema: A Systematic Review and Meta-Analysis. Dermatitis.

[127] Negrut R.L., Cote A., Feder B., Bodog F.D., Maghiar A.M. (2025). Comparative Prognostic Role of PLR and NLR in Colon Cancer: A Retrospective Analysis of Preoperative Inflammatory Markers. Medicina.

[128] Buhaș C.L., Mihalache G.C., Judea-Pusta C.T., Daina L.G., Muțiu G., Buhaș B.A., Popa A.R., Jurcă M.C., Nicoară N.D., Maghiar A.M. (2019). The Importance of the Histopathological Examination in Establishing the Diagnosis of Delayed Splenic Rupture. Report of a Case and Literature Review. Rom. J. Morphol. Embryol..

[129] Cote A., Negrut R.L., Feder B., Antal I.A., Horgos M.S., Tomescu E., Maghiar A.M. (2025). Barriers to Seeking Medical Care for Hemorrhoidal Symptoms: A Cross-Sectional Observational Study. J. Clin. Med..

[130] Rat L.A., Ghitea T.C., Maghiar A.M. (2025). Effectiveness of a Self-Esteem Enhancement Intervention Integrated into Standard CBT Protocol for Improving Quality of Life in Patients with Colorectal Cancer. Eur. J. Investig. Health Psychol. Educ..

