# Supplementary material for: Occupational Contact Dermatitis in the Post-COVID Era: From Barrier Dysfunction and Microbiome Dysbiosis to Prevention and Precision Management
Source: J Clin Med. 2026 Aug 17;15(16):6353. doi: 10.3390/jcm15166353 (PMC13513278; doi:10.3390/jcm15166353)
Supplement: Supplementary file 1 [file jcm-15-06353-s001.zip › jcm-4471286-supplementary.pdf]

Supplementary Material S1
Literature search strategy

Occupational Contact Dermatitis in the Post-COVID Era: From Barrier Dysfunction and Microbiome Dysbiosis to Prevention and Precision Management

Databases searched: PubMed/MEDLINE, the Cochrane Library (CDSR and CENTRAL), Scopus, Web of Science Core Collection

Coverage period: 1 January 2000 to 31 December 2026

Language: English

Original search completed: June 2026

Search updated during revision: August 2026

Records retrieved: 36,865 across the four databases

References cited in the review: 130

Two complementary searches were run in each database. Search A combined the condition block with an occupational-exposure block; Search B combined the condition block with a mechanism, diagnosis and intervention block. A combined search was then run to obtain the number of unique records per database.

The strings below are reproduced exactly as executed. Minor differences between them reflect the syntax accepted by each interface: mid-word wildcards are not supported in Scopus, wildcards inside quoted phrases are not supported in Web of Science, and PubMed does not apply truncation within quoted phrases, so plural forms are written out.

1. PubMed / MEDLINE

Search A — 3463 records

```
("Dermatitis, Occupational"[Mesh] OR "Dermatitis, Contact"[Mesh] OR "Dermatitis, Allergic Contact"[Mesh] OR "Dermatitis, Irritant"[Mesh] OR "occupational contact dermatitis"[tiab] OR "hand eczema"[tiab] OR "hand dermatitis"[tiab] OR "irritant contact dermatitis"[tiab] OR "allergic contact dermatitis"[tiab]) AND ("Occupational Exposure"[Mesh] OR "Health Personnel"[Mesh] OR occupational[tiab] OR "work related"[tiab] OR workplace[tiab] OR "wet work"[tiab] OR glove[tiab] OR gloves[tiab] OR "healthcare workers"[tiab] OR "health care workers"[tiab] OR hairdresser[tiab] OR hairdressers[tiab] OR metalworker[tiab] OR metalworkers[tiab] OR cleaners[tiab] OR "food handlers"[tiab]) AND (english[Filter]) AND (2000:2026[pdat])
```

Search B — 8172 records

```
(condition block as in Search A) AND ("Filaggrin Proteins"[Mesh] OR "Microbiota"[Mesh] OR "Antimicrobial Cationic Peptides"[Mesh] OR "Patch Tests"[Mesh] OR "Emollients"[Mesh] OR "skin barrier"[tiab] OR filaggrin[tiab] OR "skin microbiome"[tiab] OR dysbiosis[tiab] OR "antimicrobial peptide"[tiab] OR "antimicrobial peptides"[tiab] OR cathelicidin[tiab] OR "LL-37"[tiab] OR "patch test"[tiab] OR "patch testing"[tiab] OR prevention[tiab] OR emollient[tiab] OR emollients[tiab] OR moisturizer[tiab] OR moisturizers[tiab] OR moisturiser[tiab] OR moisturisers[tiab] OR "barrier cream"[tiab] OR "barrier creams"[tiab] OR delgocitinib[tiab] OR alitretinoin[tiab] OR dupilumab[tiab]) AND (english[Filter]) AND (2000:2026[pdat])
```

Combined (A OR B) — 9724 unique records

Combined in the PubMed Advanced search History as #1 OR #2.

2. Cochrane Library

Run in Advanced Search, single line, field set to Title Abstract Keyword. Word variations were searched. No publication-year limit was applied; the database was interrogated at Issue 8 of 12, August 2026.

Search A — 2 Cochrane Reviews, 188 records in CENTRAL

```
("occupational contact dermatitis" OR "contact dermatitis" OR "hand eczema" OR "hand dermatitis" OR "irritant contact dermatitis" OR "allergic contact dermatitis") AND (occupational OR "work related" OR workplace OR "wet work" OR glove OR gloves OR "healthcare workers" OR "health care workers" OR hairdresser OR hairdressers OR metalworker OR metalworkers OR cleaners OR "food handlers")
```

Search B — 6 Cochrane Reviews, 649 records in CENTRAL

```
(condition block as in Search A) AND ("skin barrier" OR filaggrin OR "skin microbiome" OR dysbiosis OR "antimicrobial peptide" OR "antimicrobial peptides" OR cathelicidin OR "LL-37" OR "patch test" OR "patch
```

```
testing" OR prevention OR emollient OR emollients OR moisturizer OR moisturizers OR moisturiser OR
moisturisers OR "barrier cream" OR "barrier creams" OR delgocitinib OR alitretinoin OR dupilumab)
```

Combined — 6 Cochrane Reviews, 688 records in CENTRAL, 694 in total

The combined line joined both exposure and mechanism blocks with OR. One Cochrane Clinical Answer was also retrieved. No protocols, editorials or special collections matched.

3. Scopus

Run in Advanced document search. The field code TITLE-ABS-KEY was applied to the whole expression. Publication years 2000 to 2026 and Language: English were applied as post-search limits.

Search A — 5582 documents

```
TITLE-ABS-KEY(("occupational contact dermatitis" OR "contact dermatitis" OR "hand eczema" OR "hand
dermatitis" OR "irritant contact dermatitis" OR "allergic contact dermatitis") AND (occupational OR "work
related" OR workplace OR "wet work" OR glove* OR "healthcare worker*" OR "health care worker*" OR
hairdresser* OR metalworker* OR cleaner* OR "food handler*"))
```

Search B — 15,833 documents

```
TITLE-ABS-KEY((condition block as in Search A) AND ("skin barrier" OR filaggrin OR "skin microbiome" OR
dysbiosis OR "antimicrobial peptide" OR "antimicrobial peptides" OR cathelicidin OR "patch test" OR "patch
testing" OR prevention OR emollient OR emollients OR moisturizer OR moisturizers OR moisturiser OR
moisturisers OR "barrier cream" OR "barrier creams" OR delgocitinib OR alitretinoin OR dupilumab))
```

Combined — 18,466 documents

```
TITLE-ABS-KEY(("contact dermatitis" OR "hand eczema" OR "hand dermatitis") AND (occupational OR workplace OR
"wet work" OR glove OR gloves OR hairdresser OR hairdressers OR metalworker OR metalworkers OR cleaner OR
cleaners OR "skin barrier" OR filaggrin OR microbiome OR dysbiosis OR cathelicidin OR "antimicrobial
peptide" OR "patch test" OR prevention OR emollient OR moisturizer OR "barrier cream" OR delgocitinib OR
alitretinoin OR dupilumab))
```

The condition block in the combined line was shortened to its three generic phrases. Because "contact dermatitis" is contained within "occupational contact dermatitis", "irritant contact dermatitis" and "allergic contact dermatitis", phrase matching retrieves the same set.

4. Web of Science Core Collection (all editions)

Run in Fielded Search with two rows, both set to Topic, joined with AND. Publication Date 2000-01-01 to 2026-12-31, refined by Languages: English.

Search A — 6470 documents

Row 1:

```
("occupational contact dermatitis" OR "contact dermatitis" OR "hand eczema" OR "hand dermatitis" OR
"irritant contact dermatitis" OR "allergic contact dermatitis")
```

Row 2:

```
(occupational OR "work related" OR workplace OR "wet work" OR glove* OR "healthcare worker" OR "healthcare
workers" OR "health care worker" OR "health care workers" OR hairdresser* OR metalworker* OR cleaner* OR
"food handler" OR "food handlers")
```

Without the language limit this search retrieved 8710 documents.

Search B — 3992 documents

Row 1 as above. Row 2:

```
("skin barrier" OR filaggrin OR "skin microbiome" OR dysbiosis OR "antimicrobial peptide" OR "antimicrobial
peptides" OR cathelicidin OR "LL-37" OR "patch test" OR "patch testing" OR prevention OR emollient* OR
moisturizer* OR moisturiser* OR "barrier cream" OR "barrier creams" OR delgocitinib OR alitretinoin OR
dupilumab)
```

Combined — 7981 documents

Row 1 as above. Row 2:

```
(occupational OR workplace OR "wet work" OR glove* OR hairdresser* OR metalworker* OR cleaner* OR "skin
barrier" OR filaggrin OR microbiome OR dysbiosis OR cathelicidin OR "antimicrobial peptide" OR
"antimicrobial peptides" OR "patch test" OR "patch testing" OR prevention OR emollient* OR moisturizer* OR
moisturiser* OR "barrier cream" OR delgocitinib OR alitretinoin OR dupilumab)
```

Without the language limit this search retrieved 8631 documents.

5. Summary of records retrieved

Database	Search A	Search B	Combined
**PubMed / MEDLINE**	3463	8172	9724
**Cochrane Library (CDSR + CENTRAL)**	190	655	694
**Scopus**	5582	15,833	18,466
**Web of Science Core Collection**	6470	3992	7981
**Total retrieved**			36,865

The total is the sum of the per-database combined counts and is not deduplicated across databases.

6. Selection of sources

This article is a narrative review. The records retrieved above were not screened sequentially against predefined eligibility criteria, and no dual-independent screening, formal deduplication cascade or risk-of-bias assessment was undertaken.

The search output was used to identify the candidate literature, from which sources were selected purposively according to the prioritisation criteria stated in Section 2 of the manuscript: preference for randomised controlled trials, systematic reviews and meta-analyses; large cohort and registry studies; mechanistic and translational work of recognised quality; and current clinical guidelines. Narrative reports, smaller case series and conference presentations were included selectively where they illustrated clinically important points not yet covered by higher-level evidence.

This was supplemented by hand-searching of the reference lists of key articles and current guidelines. Selection was carried out by the first author and the resulting set was reviewed and agreed by the co-authors. Clinical guidelines, regulatory documents and references identified through hand-searching or suggested during peer review entered the review outside the database searches and are not counted in the totals above.

7. Flow of identification and selection

The flow from database retrieval to the final cited set is shown as Figure 1 of the main manuscript. It is presented there as a descriptive account of identification and selection and explicitly not as a PRISMA flow diagram, for the reasons given in Section 6 above.
